# Chemical Fingerprinting of *Spiranthes spiralis* L. Methanol Seed Extract: Spectroscopic, Chromatographic, and Computational Approaches

**DOI:** 10.1002/fsn3.71531

**Published:** 2026-02-11

**Authors:** Erdi Can Aytar, Taşkın Basılı, Altevir Rossato Viana, Bengisu Şentürk, Emine İncilay Torunoğlu, Major Mabuza, Mika Sillanpää, Yasemin Özdener Kömpe

**Affiliations:** ^1^ Department of Horticulture, Faculty of Agriculture Usak University Uşak Türkiye; ^2^ Department of Chemistry, Faculty of Science Ondokuz Mayıs University Samsun Türkiye; ^3^ Department of Biochemistry and Molecular Biology Federal University of Santa Maria Santa Maria Brazil; ^4^ Department of Biology, Faculty of Science Ondokuz Mayıs University Samsun Türkiye; ^5^ Department of Medical Biochemistry, Faculty of Medicine Necmettin Erbakan University Konya Türkiye; ^6^ Department of Chemical Engineering Technology, Faculty of Engineering and the Built Environment University of Johannesburg Johannesburg Republic of South Africa; ^7^ Key Laboratory of Northwest Water Resource, Environment and Ecology, MOE Xi'an University of Architecture and Technology Xi'an China; ^8^ Institute for Nanotechnology and Water Sustainability (iNanoWS), College of Science, Engineering and Technology University of South Africa Johannesburg South Africa; ^9^ Centre of Research Impact and Outcome, Chitkara University Institute of Engineering and Technology Chitkara University Rajpura Punjab India

**Keywords:** DFT calculations, FTIR analysis, GC–MS profiling, molecular docking, orchid seed phytochemistry

## Abstract

In this study, the morphological properties, antioxidant activities, and phytochemical content of *Spiranthes spiralis* L. seed methanol extracts were characterized and analyzed in silico. Microscopic analysis revealed a fusiform seed shape characterized by prominent basal cells with thick, slanted ridges and polygonal testa structures. Fourier transform infrared spectroscopy (FTIR) identified distinct absorption bands corresponding to O–H, –CH_2_–, C=O, and other functional groups, indicating the presence of phenolic compounds, proteins, and polysaccharides. The total phenolic content was measured at 24.65 ± 1.43 mg GAE/g dry weight, while flavonoid and tannin contents were determined to be 43.98 ± 3.10 mg QE/g dry weight and 2.13 ± 0.13 mg GAE/g dry weight, respectively. Antioxidant activity, assessed via DPPH radical scavenging, yielded an IC_50_ value of 0.21 mg/mL, indicating a strong antioxidant potential associated with the phenolic content. The gas chromatography–mass spectrometry (GC–MS) analysis revealed 20 bioactive compounds, with 2,2‐dimethoxybutane and hydrazinecarbothioamide among the major constituents. Molecular docking indicated high binding affinities to the GPR52 receptor, with 4,4,6,6‐tetramethyl‐1,3‐dioxane displaying the lowest binding energy (−6.3 kcal/mol). Absorption, distribution, metabolism, excretion, and toxicity (ADMET) predictions revealed mixed toxicity profiles, as hydroxyacetic acid, hydrazide, and hydrazinecarbothioamide were flagged for potential mutagenicity. Further computational analysis, including Molecular Electrostatic Potential (MEP) and non‐covalent interaction (NCI) and reduced density gradient (RDG) mapping, supported the presence of well‐defined electrostatic regions and weak interaction zones. Additionally, the highest occupied molecular orbital (HOMO)–lowest unoccupied molecular orbital (LUMO) and global reactivity descriptors suggested that hydrazinecarbothioamide exhibits high electrophilicity and reactivity. At the same time, 2,2‐dimethoxybutane was found to be more chemically stable and less reactive. Overall, the findings emphasize the phytochemical richness and bioactive potential of *S. spiralis* seeds, offering promising perspectives for future pharmacological and biotechnological applications.

## Graphical abstract

*Spiranthes spiralis* L. seed methanol extract was morphologically and chemically characterized using microscopy, FT‐IR, GC–MS, and in silico analyses. The extract exhibited high phenolic content and strong DPPH radical scavenging activity, while GC–MS identified 22 bioactive compounds with notable binding affinity toward the GPR52 receptor. Molecular docking, ADMET, and electronic structure analyses highlighted the bioactive and pharmacological potential of *S. spiralis* seeds.

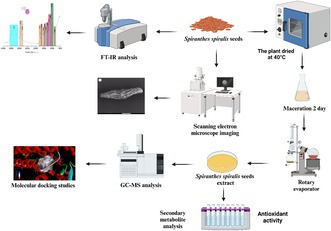

## 1 Introduction

Over the past decade, consumer preferences have played a significant role in shaping the global food supply chain. There has been a noticeable shift from synthetic to natural food ingredients, accompanied by growing interest in foods with high nutritional value. This trend has prompted the food industry to increasingly incorporate plant‐based products including fruits, vegetables, herbs, and spices (Kandyliari et al. 2023). However, the growing demand for food products, particularly fruits and vegetables, has led to the generation of considerable amounts of food waste, such as peels and seeds, although biodegradable, they pose serious environmental challenges (Chaboud and Daviron 2017; Kummu et al. 2012), including greenhouse gas emissions (primarily methane), as well as soil and water pollution. Antioxidants are compounds that neutralize free radicals within the human body. Although the body possesses its own endogenous antioxidant defense system to regulate oxidative stress, natural antioxidants derived from plant‐based diets play a crucial role in supporting this system and contribute significantly to the prevention of various diseases (Marinelli et al. 2020). Antioxidants are essential compounds that help inhibit oxidation processes. Oxidation is a chemical reaction that leads to the formation of free radicals, which in turn can trigger harmful chain reactions within cells (Rahaman et al. 2023; Sharifi‐Rad et al. 2020). Antioxidants are compounds that help eliminate these free radicals from the human body. Although the body possesses its own antioxidant defense system to manage oxidative stress, natural antioxidants obtained through plant‐based diets play a crucial supportive role and contribute significantly to the prevention of chronic diseases (Popović‐Djordjević et al. 2022).

Plants synthesize a wide array of secondary metabolites, exhibiting extensive chemical diversity and a range of biological activities (Khammassi et al. 2023). The production of these compounds can vary not only between species but also among different organs of the same plant. Furthermore, factors such as developmental stage, climate, and soil conditions (edaphic factors) significantly influence their biosynthesis (Kouki et al. 2022). Although oxidative stress poses serious risks to human health, the use of synthetic antioxidant agents has not always proven effective (Serwecińska 2020). As a result, exploring natural sources for novel bioactive compounds is increasingly recognized as a more sustainable and promising approach, both for health promotion and environmental protection (Ndhlala et al. 2022).

Orchids are distributed globally, except for desert and polar regions. Each year, approximately 200 new orchid species are identified, primarily in tropical areas (Kumar et al. 2022). Beyond their ornamental appeal, orchids are recognized for their pharmacological potential. Many medicinal orchid species are rich in phytochemicals and exhibit a broad spectrum of biological activities, including antipyretic, anti‐rheumatic, anti‐inflammatory, anticarcinogenic, anticonvulsant, diuretic, neuroprotective, sedative, anti‐aging, antiseptic, hypoglycemic, antitumor, antimicrobial, antiviral, and immunomodulatory effects (Bhattacharyya et al. 2019; Musharof Hossain 2011).

Although orchid seeds are microscopic in size, they are produced in remarkably high numbers; a single capsule can contain between 2 and 2 million seeds (Arditti and Ghani 2000). These seeds are among the smallest in the plant kingdom. Their minute, dust‐like structure presents challenges for studying seed dispersal, germination, and seedling development (Rasmussen et al. 2015). The genus *Spiranthes* comprises approximately 40 species worldwide, with four species *Spiranthes aestivalis*, *Spiranthes romanzoffiana* , *Spiranthes spiralis* L., and *Spiranthes sinensis* native to Europe. While morphologically well‐characterized, the genus is primarily centred in North and Central America. *S. spiralis* shows minimal morphological variation across its geographical distribution (Pridgeon et al. 2001).

Different extraction techniques rely on distinct physical principles to isolate target compounds efficiently. Microwave‐assisted extraction (MAE) applies electromagnetic radiation to induce dipolar rotation and ionic conduction, generating heat within the sample matrix. Ultrasonic‐assisted extraction (UAE) employs low‐frequency sound waves to create high‐energy cavitation bubbles that disrupt cellular structures and enhance mass transfer. Pressurized liquid extraction (PLE) utilizes elevated temperatures and pressures to maintain solvents in a liquid state above their boiling points, thereby enhancing solvent penetration and solubilization of analytes. Supercritical fluid extraction (SFE), most commonly utilizing carbon dioxide, operates at conditions above the fluid's critical temperature and pressure to achieve enhanced solvating power and selective compound recovery (Cacique et al. 2020).

Although the extraction techniques offer high efficiency, they may present limitations related to cost, operational complexity, or compound stability. For example, MAE may induce lipid oxidation within the sample matrix due to the intense localized heating. Similarly, ultrasonic‐assisted extraction can negatively impact the integrity of thermolabile or sensitive compounds, depending on the energy intensity and exposure duration. Therefore, careful optimization and preliminary studies are essential before applying such advanced methods. In contrast, conventional extraction techniques such as maceration, stir‐assisted maceration, and Soxhlet extraction remain widely used due to their simplicity, accessibility, and minimal equipment requirements (Alara et al. 2019). Maceration is a conventional extraction method based on solid–liquid separation, typically using organic solvents or water to isolate bioactive compounds. Commonly employed solvents for extracting phenolic constituents include methanol, ethanol, water, or their combinations. However, a standardized solvent system for optimal phenolic extraction from plant matrices has yet to be established, as efficiency varies depending on the plant species and the polarity of the compounds. The performance of the maceration process can be further improved by optimizing parameters such as homogenization, extraction time, and temperature (Gori et al. 2021; Irfan et al. 2022).

Computational chemistry is a powerful area based on simulations and quantum mechanical models that enable the prediction and interpretation of chemical behavior at the molecular level (Zhuo 2024). Among these methods, density functional theory (DFT) stands out due to its highly accurate description of the electronic structure, reactivity, and stability of chemical systems at a reasonable computational cost (Kreuter 2007). DFT calculations are widely used in pharmaceutical chemistry, materials science, and natural product research, primarily in the study of boundary molecular orbitals, global reactivity descriptors, and electrostatic surface distributions (Fifen et al. 2011).

In recent years, the integration of non‐covalent interaction analyses, such as molecular electrostatic potential (MEP) mapping and the reduced density gradient (RDG) method, has led to a deeper understanding of intermolecular forces and reactive sites. These techniques provide important insights into molecular recognition and biological activity by visualizing weak interactions such as hydrogen bonds, van der Waals forces and steric repulsions (Boukabcha et al. 2023; Johnson et al. 2010).

*Spiranthes spiralis*, a species belonging to the Orchidaceae, has attracted attention in recent years due to its potential to carry biologically active compounds. However, a comprehensive density functional theory (DFT)‐based computational study focusing on the major phytochemicals present in the seeds of this species is currently unavailable. In this study, 2,2‐dimethoxybutane, hydrazinecarbothioamide, di‐sec‐butyl ether, and hydroxyacetic acid hydrazide molecules, which are the main compounds identified by gas chromatography–mass spectrometry (GC–MS) analysis in *S. spiralis* seed extract, were theoretically evaluated using the DFT method. These compounds are believed to significantly contribute to the antioxidant and biological activity of the plant.

## 2 Materials and Methods

### 2.1 Collection of Seed Samples

The aboveground parts of *S. spiralis* L. were collected during the maturation period in November 2024 from the campus of Ondokuz Mayıs University, Samsun (41°22′01″ N 36°11′10″ E) (OMUB‐7729), and the plant material was taxonomically identified by Prof. Dr. Yasemin Özdener Kömpe.

### 2.2 Scanning Electron Microscopy (SEM) Analysis

Dry seeds were carefully isolated and mounted on aluminium stubs using double‐sided carbon adhesive tape before scanning electron microscopy analysis. To enhance electrical conductivity and obtain clear images, the samples were coated with a thin layer of gold–palladium alloy (approximately 12.5–15 nm) using a sputter coater (SC7620). Scanning electron microscopy (SEM) observations were conducted using a JEOL JMS‐7001F microscope with an accelerating voltage ranging from 5 to 15 kV. High‐resolution images were acquired to evaluate morphological characteristics, including seed shape, surface ornamentation of the testa, and the structural features of cell wall boundaries (Gamarra et al. 2012).

### 2.3 Fourier Transform Infrared (FTIR) Spectroscopy Analysis

Fourier transform infrared (FTIR) spectroscopy analyses were carried out using a PERKIN ELMER Spectrum TWO spectrometer (Perkin Elmer Inc., Norwalk, CT, USA) equipped with a diamond attenuated total reflectance (ATR) accessory and a lithium tantalate (LiTaO_3_) detector. Spectra were recorded in the range of 4000–400 cm^−1^ with a spectral resolution of 4 cm^−1^. After data acquisition, the resulting spectra were processed, and the characteristic absorption bands were interpreted using PerkinElmer Spectrum One FTIR software.

### 2.4 Preparation of Methanol Extracts

The dry seeds of *S. spiralis* were ground into a fine powder using a laboratory blender. Then, 0.1 g of the powdered seeds was mixed with 10 mL of 80% methanol and left at 35°C for 24 h with occasional shaking. The mixture was subsequently filtered using Whatman filter paper (No. 1), and the filtrate was stored at 4°C until use for further analyses.

### 2.5 Spectrophotometric Evaluation of Secondary Metabolites and Antioxidant Activity

The quantitative determination of secondary metabolites, including phenolics, flavonoids, flavanols, tannins, and proanthocyanidins, together with an assessment of their antioxidant activity, was carried out using validated and optimized spectrophotometric methods.

The total phenolic content was determined by reacting 200 μL of the seed extract (1 mg/mL) with 200 μL of Folin–Ciocalteu reagent, which had been previously diluted with distilled water (1:1, v/v). Following a 3‐min incubation at room temperature, 1 mL of 2% Na_2_CO_3_ solution was added, and the mixture was incubated in the dark for 60 min. Absorbance was recorded at 760 nm, and results were expressed as milligrams of gallic acid equivalents per gram of dry weight (mg GAE/g dry weight) (Singleton and Rossi 1965).

Total flavonoid content was measured by mixing 1 mL of the sample with 6.4 mL of distilled water, followed by the addition of 0.3 mL of 5% NaNO_2_ and 0.3 mL of 10% AlCl_3_ with appropriate incubation intervals. Subsequently, 2 mL of 1 M NaOH was added, and absorbance was measured at 510 nm. The flavonoid content was expressed as milligrams of quercetin equivalents per gram of dry weight (mg QE/g dry weight) (Osuna‐Ruiz et al. 2016).

Flavanol concentration was evaluated by combining 1 mL of the extract with 2 mL of 2% AlCl_3_ and 3 mL of 5% sodium acetate. After incubation in the dark for 30 min, absorbance was measured at 415 nm, and results were reported as mg QE/g dry weight (Mahmoudi et al. 2023).

Tannin content was assessed by mixing 100 μL of the sample, diluted fivefold, with 500 μL of diluted Folin reagent and 2 mL of 4% Na_2_CO_3_. The mixture was incubated in the dark for 30 min, after which absorbance was read at 760 nm. Tannin levels were expressed as mg GAE/g dry weight (Lou et al. 2014).

Proanthocyanidin content was determined using the butanol–HCl method. Briefly, 500 μL of diluted extract (1:5, v/v) was mixed with 3 mL of n‐butanol: HCl (95:5, v/v) and 100 μL of 2% ferric ammonium sulfate prepared in 2 N HCl. The reaction mixture was heated in a boiling water bath for 60 min. After cooling, absorbance was measured at 550 nm, and results were expressed as milligrams of catechin equivalents per gram of dry weight (mg CAE/g dry weight) (Porter et al. 1985).

The antioxidant capacity of the extracts was additionally evaluated using the 2,2‐Diphenyl‐1‐picrylhydrazyl (DPPH) activity. In this essay, 1 mL of extract at different concentrations was mixed with 1 mL of 0.1 mM DPPH solution prepared in methanol. The reaction was allowed to proceed for 30 min in the dark at room temperature, and absorbance was subsequently measured at 517 nm (Braca et al. 2001).

### 2.6 Gas Chromatography–Mass Spectrometry (GC–MS) Analysis

Methanol extracts obtained from *S. spiralis* seeds were diluted at a 1:100 ratio and subsequently centrifuged at 3500 rpm for 10 min. Following centrifugation, the clear supernatant was carefully collected and transferred into 1.5 mL vials for further analysis. GC–MS measurements were carried out using a SHIMADZU GC–MS‐QP2010 instrument equipped with an Rxi‐5MS capillary column. The analysis was conducted under electron ionization mode at 70 eV, with helium employed as the carrier gas at a constant flow rate of 1 mL/min. Samples were injected in a volume of 1.5 μL using a split ratio of 10:1. The oven temperature program was set to increase from 70°C to 250°C, resulting in a total analysis time of 56.7 min. Identification of the detected compounds was achieved by comparison of mass spectra with those in the NIST library (Aytar and Aydın 2024).

### 2.7 Molecular Docking Studies

For the molecular docking analysis, the major phytochemical constituents identified in the seed extracts were retrieved from the PubChem database. The structures, initially provided in SDF format, were converted into PDB files using Discovery Studio Visualizer. Rotatable bonds were defined, and torsional degrees of freedom were optimized to ensure ligand flexibility during docking. The prepared ligands were subsequently imported into PyRx, where AutoDock Vina was used to generate the corresponding PDBQT files.

Regarding receptor preparation, the crystal structures of the selected target proteins, including the ligand‐free GPR52 structure fused with flavodoxin (PDB ID: 6LI1), were downloaded from the RCSB Protein Data Bank. All crystallographic water molecules were removed, non‐standard residues were corrected, and Gasteiger charges were assigned to standardize the protein structures. The finalized receptors were then converted to PDBQT format using AutoDock Tools (version 4.2). Docking simulations were performed with AutoDock Vina, during which ligand geometries, charges, and torsional parameters were optimized. For each ligand, multiple binding conformations were generated, and the pose exhibiting the lowest binding energy was selected for detailed analysis. The resulting protein–ligand complexes were visualized, and their interaction patterns were evaluated using Discovery Studio Visualizer to determine binding modes and key molecular interactions (Trott and Olson 2010).

### 2.8 Computational Details

In this work, all computational procedures, including molecular geometry optimization and the evaluation of electronic parameters such as highest occupied molecular orbital (HOMO)–lowest unoccupied molecular orbital (LUMO) energy gaps, global reactivity descriptors, and molecular electrostatic potential distributions, were conducted using the Gaussian 09 software package. DFT calculations were performed employing the B3LYP functional in combination with the 6‐31G(d,p) basis set, which provides reliable estimations of molecular energies and electronic properties. All theoretical calculations were executed on the Arf computing cluster within the Turkish National Science e‐Infrastructure (TRUBA).

To investigate weak intermolecular interactions, NCI analyses were performed using RDG calculations with the Multiwfn program. Visualization of optimized molecular geometries and computational results was performed using GaussView 05 and VMD (Visual Molecular Dynamics). Additionally, MarvinSketch 23.17 (ChemAxon) was employed for molecular structure drawing and representation (Humphrey et al. 1996).

### 2.9 Predict Absorption, Distribution, Metabolism, Excretion, and Toxicity (ADMET) Studies

Prediction of pharmaceutical and pharmacokinetic properties was conducted using the freely accessible pkCSM online platform for pharmacokinetics modeling. The in silico evaluation of the major compound focused on its absorption, distribution, metabolism, excretion, and toxicity (ADMET) characteristics as well as potential toxicity profiles. The analyzed parameters comprised mutagenicity (AMES test), maximum tolerated dose, inhibition potential toward the human ether‐a‐go‐go related gene (hERG I and II) channels, acute oral toxicity in rats (LD_50_), chronic oral toxicity in rats (LOAEL), hepatotoxic effects, and skin sensitization potential.

### 2.10 Statistical Analysis

For fatty acid profiling and biological activity assessments, three independent extracts were prepared from each powdered seed sample. All experimental data were subsequently analyzed using IBM SPSS Statistics version 22.

## 3 Results and Discussion

### 3.1 Morphological Characterization of Seeds

**FIGURE 1:**
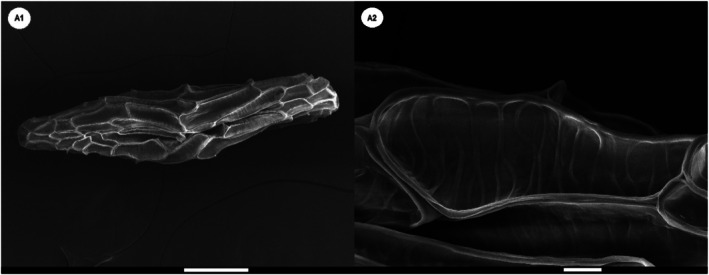
Scanning electron micrographs of *Spiranthes spiralis* seeds. (A1) Overview of the whole seed; (A2) Surface morphology of testa cells. The scale bar represents 100 μm in image A1 and 10 μm in image A2.

The seeds of *S. spiralis* exhibit a distinctly fusiform morphology. The basal cells are marked by prominent, thickened ridges arranged in a slanted and spaced pattern. Testa cells appear polygonal, isodiametric, and elongated in shape. Additionally, all seeds display a pronounced sunken line along their longitudinal anticlinal walls, indicating structural uniformity in this feature (Figure 1).

### 3.2 Fourier Transform İnfrared Spectroscopy (FTIR) Analysis of *S. spiralis* Seeds

**FIGURE 2:**
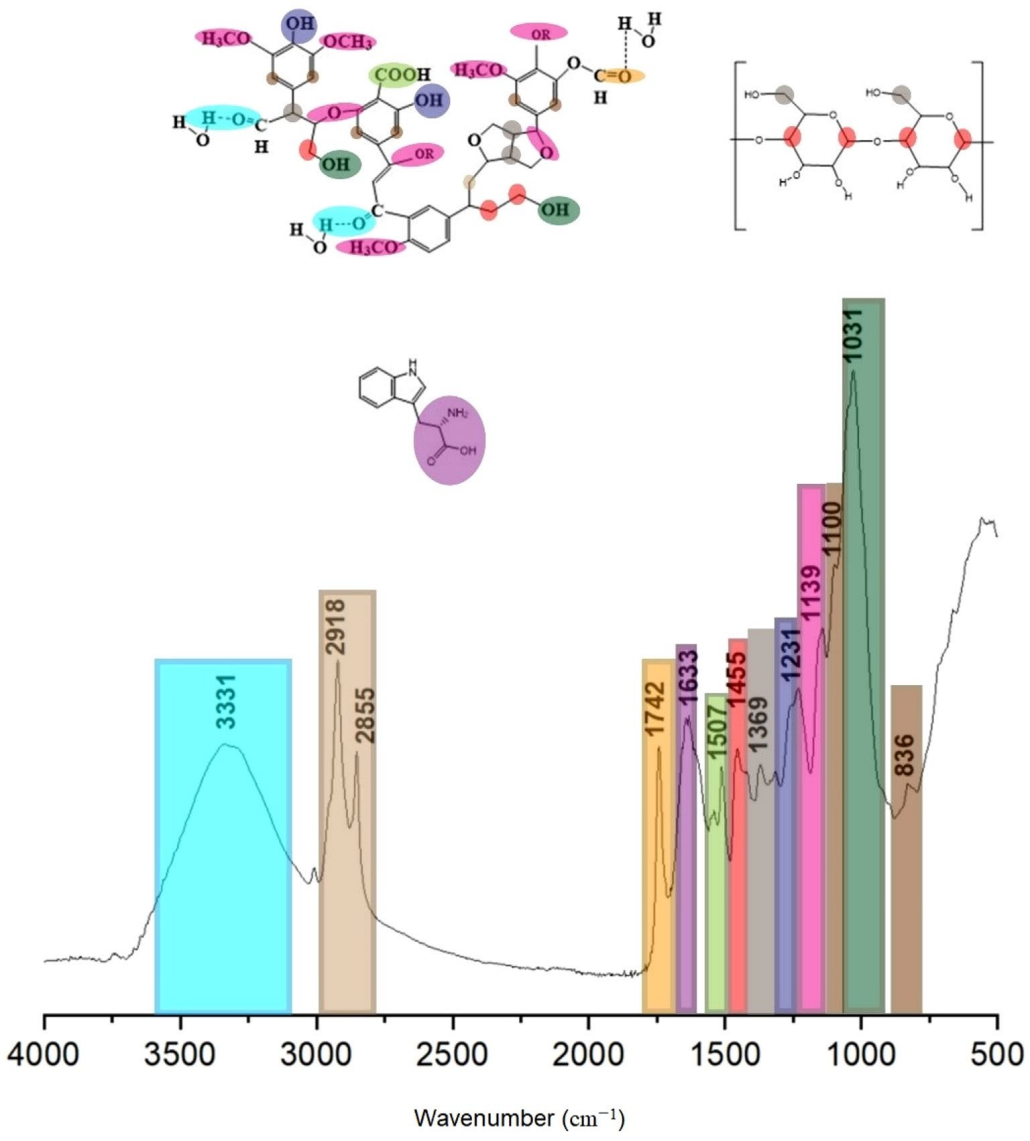
FTIR spectrum of *Spiranthes spiralis* seeds illustrating major functional groups and associated bond vibrations.

Figure 2 presents the Fourier transform infrared spectroscopy (FTIR) spectrum of *S. spiralis* seeds. The analysis revealed characteristic absorption bands at various wavenumbers. A broad peak at 3331 cm^−1^ corresponds to O–H stretching vibrations, while bands at 2918 and 2855 cm^−1^ are associated with –CH_2_– stretching. The peak observed at 1742 cm^−1^ is indicative of C=O stretching vibrations related to carboxyl and acetyl groups, commonly found in hemicellulose. A band at 1633 cm^−1^ is attributed to C=O stretching from amide groups, reflecting protein presence. Additional bands include 1507 cm^−1^, corresponding to COO^−^ stretching; 1455 cm^−1^, related to C–H deformation in cellulose; and 1369 cm^−1^, linked to asymmetric CH_2_ bending in hemicellulose. Further peaks were detected at 1231 cm^−1^ (O–H bending in cellulose and lignin), 1139 cm^−1^ (C–H stretching of phenolic acids), and 1100 cm^−1^ (aromatic C–H deformation in lignin syringyl units). At last, the peak at 1031 cm^−1^ represents O–H stretching of primary alcohols in polysaccharides, cellulose, and lignin, along with C–H out‐of‐plane deformation in aromatic lignin structures.

The obtained spectra showed peaks at various wavenumbers: 3331, 2918, and 2855 cm^−1^, corresponding to the characteristic O–H stretching vibration (Hospodarova et al. 2018). The peaks at 2918 and 2855 cm^−1^ are attributed to –CH_2_– stretching Capobianco et al. (2023) while the peak at 1742 cm^−1^ indicates the C=O stretching vibration of carboxyl and acetyl groups in hemicellulose (Poletto et al. 2011). The band at 1633 cm^−1^ represents the C=O stretch vibrations of the amide groups in proteins (Dominic et al. 2022). Additionally, peaks at 1507 cm^−1^ are related to COO– stretching (Droussi et al. 2009), 1455 cm^−1^ to C–H plane deformation in cellulose Szymańska‐Chargot et al. (2022), and 1369 cm^−1^ to asymmetric CH_2_ bending in hemicellulose (Veisi et al. 2023). Furthermore, the peaks at 1231 cm^−1^ indicate in‐plane OH bending in cellulose and lignin (Kayabaş and Yildirim 2022), 1139 cm^−1^ represents phenolic acids' C–H stretching Soukup et al. (2020), 1100 cm^−1^ corresponds to the lignin aromatic C–H deformation of syringyl units Lehto et al. (2018), and 1031 cm^−1^ is related to polysaccharides, cellulose, and lignin O–H stretching of primary alcohols Narendhran and Sivaraj (2016), and aromatic lignin, C–H out of plane deformation (Gilca et al. 2014).

### 3.3 Antioxidant and Phenolic Profile of *S. spiralis* Seed

The results were expressed as mg GAE/g dry weight based on the calibration curve derived from gallic acid standards. The total phenolic content of the extract was calculated as 24.65 ± 1.43 mg GAE/g dry weight. Similarly, the total flavonoid content, estimated using the quercetin calibration curve, was found to be 43.98 ± 3.10 mg QE/g extract. Flavanol content, also assessed using quercetin as the reference compound, was determined to be 37.09 ± 1.11 mg QE/g dry weight. The total tannin content, calculated relative to gallic acid equivalents, was 2.13 ± 0.13 mg GAE/g dry weight. Furthermore, proanthocyanidin content was quantified based on a catechin standard curve and found to be 138.78 ± 20.46 mg CAE/g dry weight. These data suggest that *S. spiralis* seeds possess a considerable concentration of phenolic constituents, as outlined in Table 1.

**TABLE 1:** Bioactive compound content and antioxidant activity of *Spiranthes spiralis* seed extract.

Plant name	DPPH (IC_50_ mg/mL)	Total flavonol compound (mg QE/g dry weight)	Total flavonoid compound (mg QE/g dry weight)	Total phenolic compound (mg GAE/g dry weight)	Total proanthocyanidin content (mg CAE/g extract)	Total tannin content (mg GAE/g dry weight)
*Spiranthes spiralis* seed	0.21 ± 0.02	37.09 ± 1.11	43.98 ± 3.10	24.65 ± 1.43	138.78 ± 20.46	2.13 ± 0.13

*Note:* Values are presented as mean ± SD (*n* = 3). Positive control: ascorbic acid (IC_50_ = 0.005 ± 0.001 mg/mL).

In addition, Table 1 displays the DPPH free radical scavenging activity of the seed extract, with an IC_50_ value of 0.21 mg/mL, indicating strong antioxidant potential in comparison to the reference compound, ascorbic acid. The observed antioxidant capacity is consistent with the high phenolic content, and a notable positive relationship was identified between total phenolic levels and radical scavenging activity, implying that these compounds are the major contributors to the extract's antioxidant effect.

Jothy et al. (2011) also calculated the IC_50_ value of *Cassia fistula* seed extract using the DPPH radical scavenging method as 11.07 mg/mL. In comparison to *S. spiralis* seed extract, the antioxidant activity of Indian laburnum seeds was found to be lower. Furthermore, Junaid et al. (2013) determined the IC_50_ value of dried *Lagerstroemia speciosa* seeds' methanol extract against DPPH radical as 9.63 ± 0.20 μg/mL. These results demonstrate that *L. speciosa* seeds have higher antioxidant activity than *S. spiralis* seeds.

The data of *S. spiralis* , along with seed extracts of other plants collected from Turkey, reveal differences in terms of phenolic content. The total phenolic content of *S. spiralis* seed extract was determined as 24.65 ± 1.43 mg GAE/g dry weight, which is a considerably high value compared to other plants' seed extracts. The total phenolic content in seed extracts of some plants ( *Juniperus oxycedrus* ssp., *Rhus coriaceae*, *Humulus lupulus* , *Umbelliferae foeniculum*, *Nigella sativa* L., *Coriandrum sativum*, *Linum usitatissimum* ) collected from Turkey varies between 1.27 and 37.20 mg GAE/g dry weight (Yemis et al. 2008). Phenolic compounds constitute the main class of plant secondary metabolites commonly found in fruits and vegetables. Due to their effective antioxidant properties, these compounds offer numerous health benefits to humans. Polyphenolic compounds can protect cellular components against damage caused by free radicals and may help prevent degenerative diseases such as cancer and cardiovascular diseases. They encompass more than 4500 compounds, including subgroups like flavonoids, anthocyanins, proanthocyanidins, and isoflavonoids. These compounds play significant roles in plant defense mechanisms and act as signaling molecules in plant–microorganism interactions, promoting beneficial relationships between plants, soil, and microorganisms (Bhattacharyya et al. 2014). For instance, arbuscular mycorrhizal fungi can enhance pre‐symbiotic growth and show beneficial effects during fungal sporulation and initial growth stages. Therefore, flavonoids play a crucial role in plant adaptation to environmental conditions and participation in mutual beneficial interactions (Cazar et al. 2023; Tlili et al. 2014; Zhuang et al. 2023).

*Spiranthes spiralis* seeds have a low tannin content. Other plants may have higher amounts of tannins. Tannins may contribute to the free radical scavenging activity and antioxidant properties of plant‐based extracts. However, *S. spiralis* seeds are not particularly rich in tannins. For instance, in comparison to seed extracts, pea extracts used in our study showed higher tannin content, measuring 62.7 and 55.7 mg CAE/100 g dry weight in methanol and ethanol, respectively (Natta et al. 2022). In our study, *S. spiralis* seeds were found to have a low tannin content, while other plants may have higher amounts of tannins. On the other hand, *S. spiralis* seeds are rich in proanthocyanidins, with a content of 138.78 ± 20.46 (Dulyanska et al. 2022) have a higher proanthocyanidin content compared to sweet cherry seeds, which range from 4.70 ± 0.81 to 22.5 ± 1.86 mg CAE/100 g dry weight. Despite the low tannin content in *S. spiralis* seeds, their high antioxidant activity suggests that other components may play a significant role in antioxidant efficacy.

In conclusion, despite the low tannin content, *S. spiralis* seeds exhibit high antioxidant activity, indicating the potential contribution of other compounds to their antioxidant efficacy. Additionally, variations in phenolic content among seed extracts of plants collected from Turkey were observed, and *S. spiralis* seed extract showed notably high phenolic content compared to others.

### 3.4 Seed Metabolite Profiling Using GC–MS Technique

The methanol extract of *S. spiralis* seeds was analyzed to identify its bioactive phytochemical constituents. Detailed information on the retention time (RT), molecular formula, molecular weight (MW), and relative concentration (expressed as % peak area) of 20 detected compounds is provided in Table 2. Among the identified constituents, the most abundant were 2,2‐dimethoxybutane (18.02%), hydrazinecarbothioamide (9.97%), di‐sec‐butyl ether (6.64%), hydroxyacetic acid, hydrazide (6.39%), and 4,4,6,6‐tetramethyl‐1,3‐dioxane (5.06%).

**TABLE 2:** Bioactive compounds identified in the methanol extract of *Spiranthes spiralis* seeds via GC–MS analysis.

No	RT (min)	Name of the compound	Molecular formula	Molecular weight	Peak area %
1	3.060	Hydroxyacetic acid, hydrazide	C_6_H_6_N_2_O_2_	90.80	6.39
2	3.085	2‐amino‐3‐hydroxypropanamide	C_3_H_8_N_2_O_2_	148.23	1.04
3	3.187	d3‐Glycine	C_2_H_5_NO_2_	78.09	4.26
4	3.393	4,4,6,6‐Tetramethyl‐1,3‐dioxane	C_8_H_16_O_2_	144.21	5.06
5	3.460	Hydrazinecarbothioamide	CH_5_N_3_S	91.135	9.97
6	3.605	Carbonic dihydrazide	CH_6_N_4_O	90.09	3.85
7	5.025	2,2‐Dimethoxybutane	C_6_H_14_O_2_	118.17	18.02
8	7.168	3,3‐Dimethoxy‐2‐butanone	C_6_H_12_O_3_	132.16	3.16
9	7.248	Butyl 2‐(2‐(2‐butoxyethoxy) ethoxy) acetate	C_10_H_20_O_4_	204.26	3.00
10	7.314	Di‐sec‐butyl ether	C_8_H_18_O	130.23	6.64
11	10.508	Decane	C_10_H_22_	142.28	4.38
12	10.805	Aziridine, 1‐(methoxymethyl)‐	C_4_H_9_NO	87.12	0.67
13	11.112	1‐Hexanol, 2‐ethyl‐	C_8_H_18_O	130.22	0.36
14	12.386	Undecane	C_11_H_24_	156.31	1.69
15	14.676	Benzothiazole	C_7_H_5_NS	135.19	1.06
16	17.710	1H‐Benzocycloheptene, 2,4a,5,6,7,8,9,9a‐octahydro‐3,5,5‐trimethyl‐9‐methylene‐, (4aS‐cis)‐	C_15_H_24_	204.35	2.22
17	34.982	Hexadecanoic acid, methyl ester	C_17_H_34_O_2_	270.45	1.02
18	42.415	9, 12‐Octadecadienoic acid (Z,Z)‐, methyl ester	C_19_H_34_O_2_	294.47	1.83
19	42.696	9‐Octadecenoic acid (Z)‐, methyl ester	C_19_H_36_O_2_	294.48	0.86
20	43.874	Methyl stearate	C_19_H_38_O_2_	298.5	1.23

There are several examples of GC–MS studies conducted to determine the biochemical components of Orchidaceae species. In a distinct and interesting study, Manzo et al. (2014) analyzed the volatile compounds of three different orchid species from Italian populations using solid‐phase microextraction (SPME) combined with MS‐linked GC. They identified hydrocarbons, aldehydes, alcohols, and terpenes as the main components of orchid fragrances. Although it is generally known that tropical orchid species contain various phytochemicals believed to provide biological activities, terrestrial orchid species have not been adequately explored in terms of antioxidant activity (Jakubska‐Busse et al. 2014). Therefore, the evaluation of seed extracts in terms of this characteristic in this study will also make a significant contribution to the field of pharmacology.

Numerous compounds found in plants possess toxic properties, which may contribute to the limited toxicity reported in this extract. Shahar et al. (2023), in their study, previously reported the toxicity of unbranched (decanes and undecanes) paraffins. Additionally, branched ether 2,2‐dimethoxybutane has been shown to exhibit toxic effects and is also found to be toxic to microbial membranes (Sani et al. 2015). However, it has been demonstrated that 2,2‐dimethoxybutane possesses various therapeutic potentials, making it a valuable feature for the development of novel drugs.

As a result, 2,2‐dimethoxybutane, a major compound found in *S. spiralis* seeds, exhibits high biological properties, rendering it suitable for the development of functional foods with enhanced nutritional content and natural product‐based drugs. Consequently, the significant biological attributes of these plants can be explored for the development of novel drugs and functional foods with improved nutritional profiles. Therefore, further research on the potential and biological properties of these plants is crucial.

Hydrazinecarbothioamides, another significant compound found in *S. spiralis* seeds, are considered versatile biological promoters, facilitated by nitrogen and sulfur donors in various coordination modes. These compounds are integrated with multiple molecules exhibiting diverse biological activities, and their potential bio‐properties are dependent on the main aldehyde or ketone connections (Aly et al. 2010). In addition to their antitumor and antiprotozoal properties, they also possess antiviral effects (Grover and Kini 2006). Moreover, their parasiticide actions against *Plasmodium falciparum* and *Trypanosoma cruzi* make them important pharmaceutical agents for malaria and Chagas disease (Du et al. 2002; Greenbaum et al. 2004). Furthermore, it has been observed that the complexation of hydrazine carbothioamide with transition elements, especially iron and copper, enhances their bioactive properties compared to the non‐complexed form (Easmon et al. 2001).

### 3.5 In Silico Binding Affinities of Selected Seed Compounds to Target Proteins and ADMET

**FIGURE 3:**
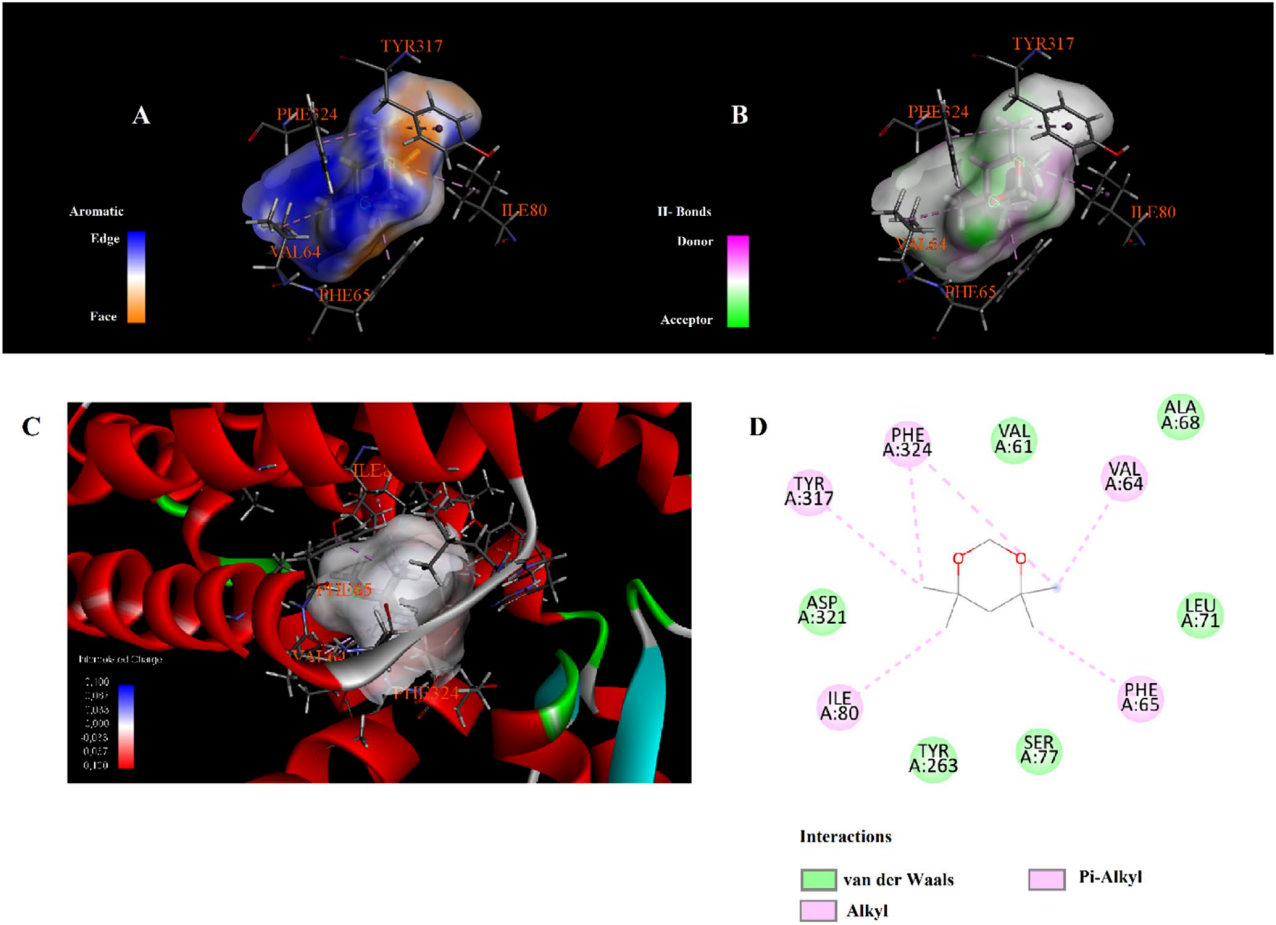
Interaction diagram of 4,4,6,6‐tetramethyl‐1,3‐dioxane with 6LI1 proteins in terms of (A) aromatic, (B) H‐bonds, (C) 3D structure, and (D) 2D interpolated charge.

**FIGURE 4:**
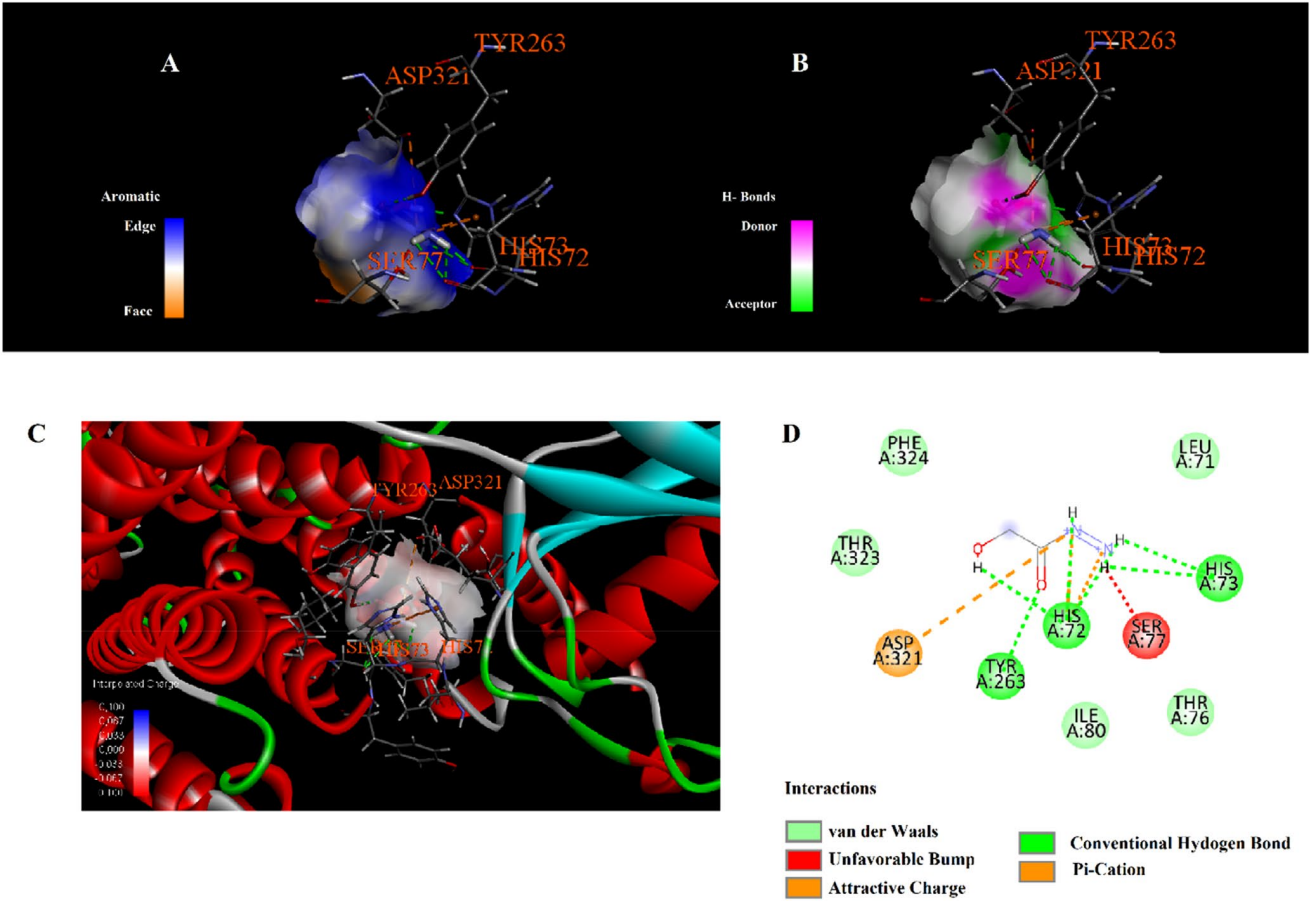
Interaction diagram of hydroxyacetic acid, hydrazide with 6LI1 proteins in terms of (A) aromatic, (B) H‐bonds, (C) 3D structure, and (D) 2D interpolated charge.

**FIGURE 5:**
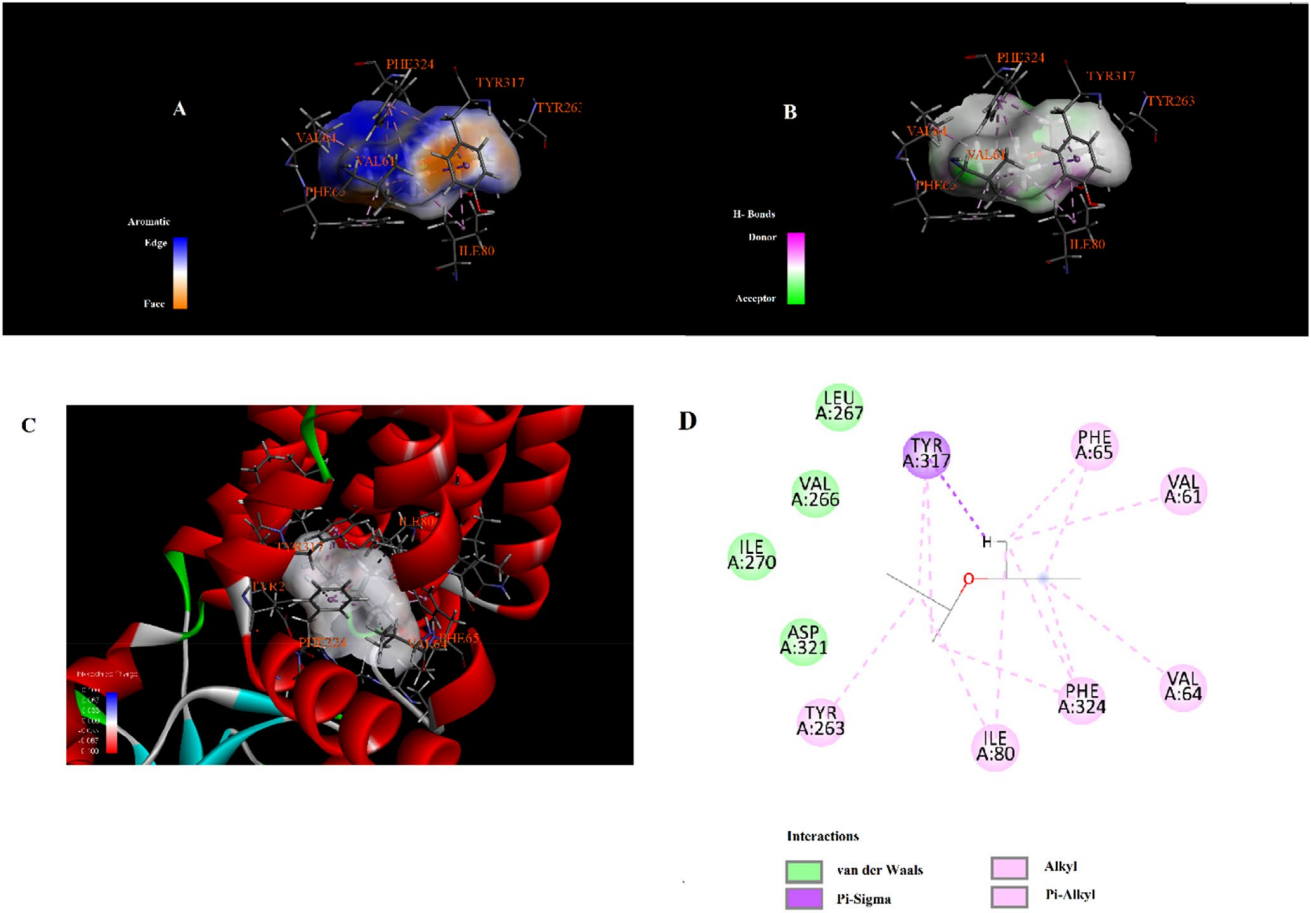
Interaction diagram of di‐sec‐butyl ether with 6LI1 proteins in terms of (A) aromatic, (B) H‐bonds, (C) 3D structure, and (D) 2D interpolated charge.

**FIGURE 6:**
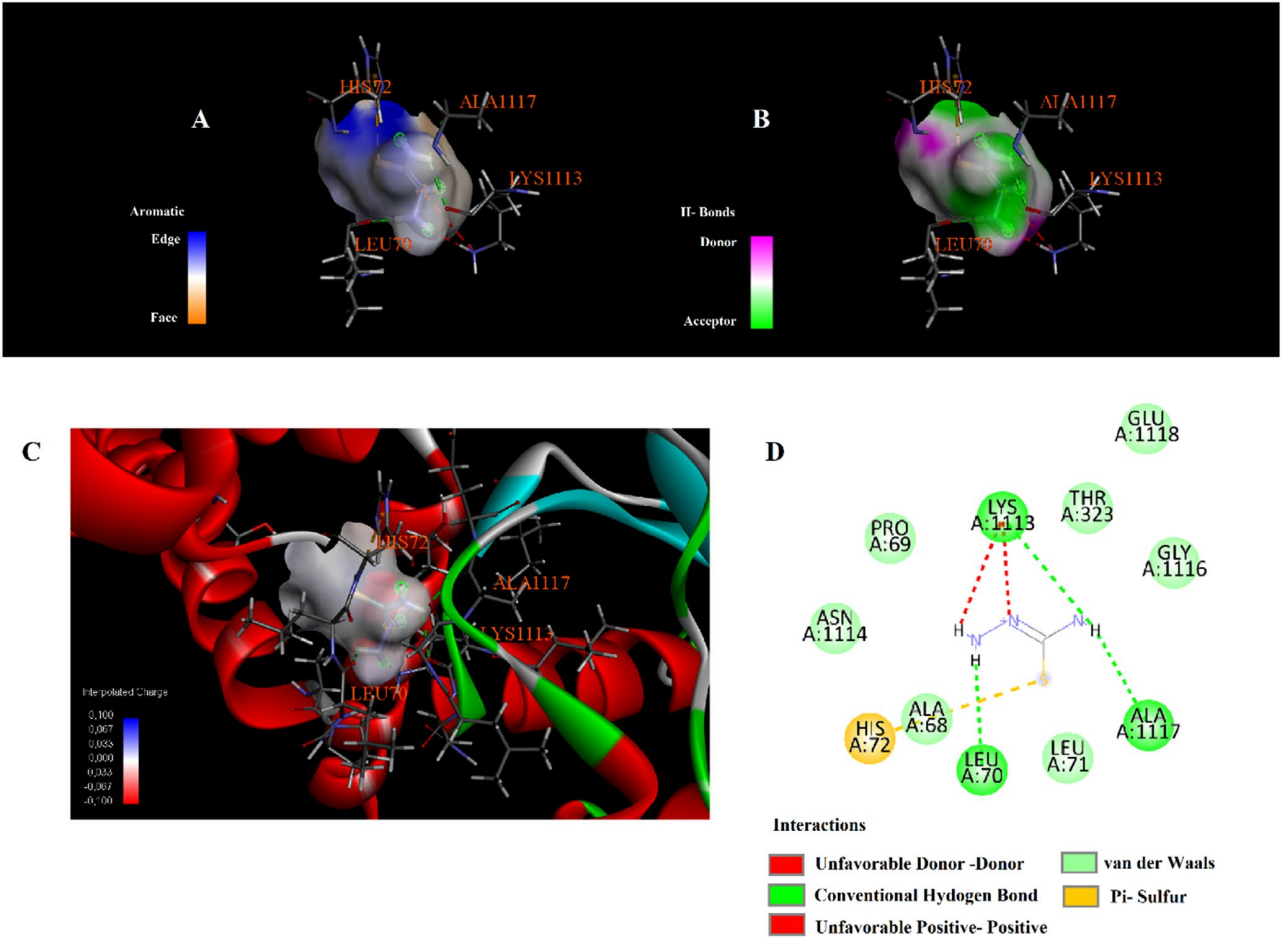
Interaction diagram of hydrazinecarbothioamide with 6LI1 proteins in terms of (A) aromatic, (B) H‐bonds, (C) 3D structure, and (D) 2D interpolated charge.

**FIGURE 7:**
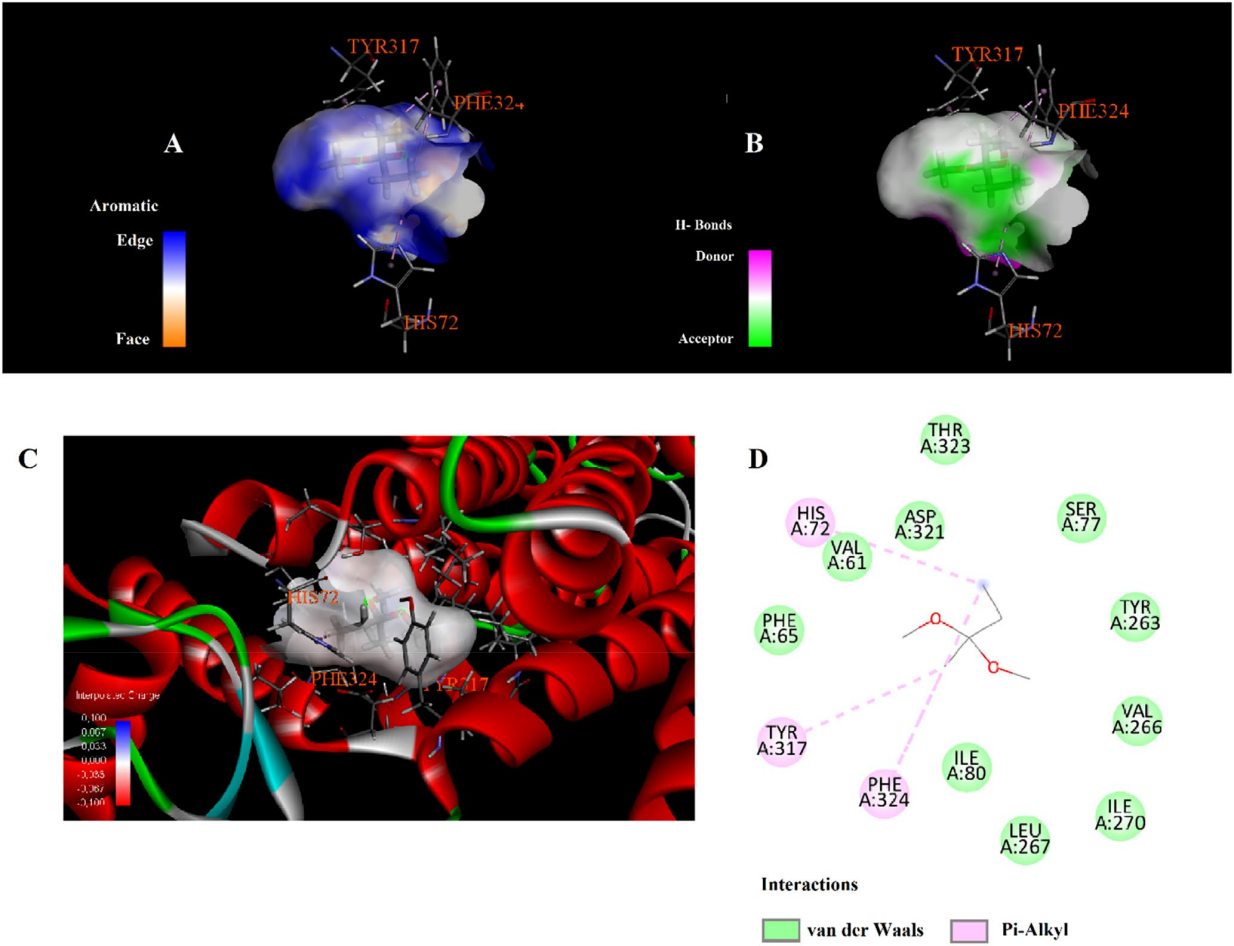
Interaction diagram of 2,2‐dimethoxybutane with 6LI1 proteins in terms of (A) aromatic, (B) H‐bonds, (C) 3D structure, and (D) 2D interpolated charge.

**FIGURE 8:**
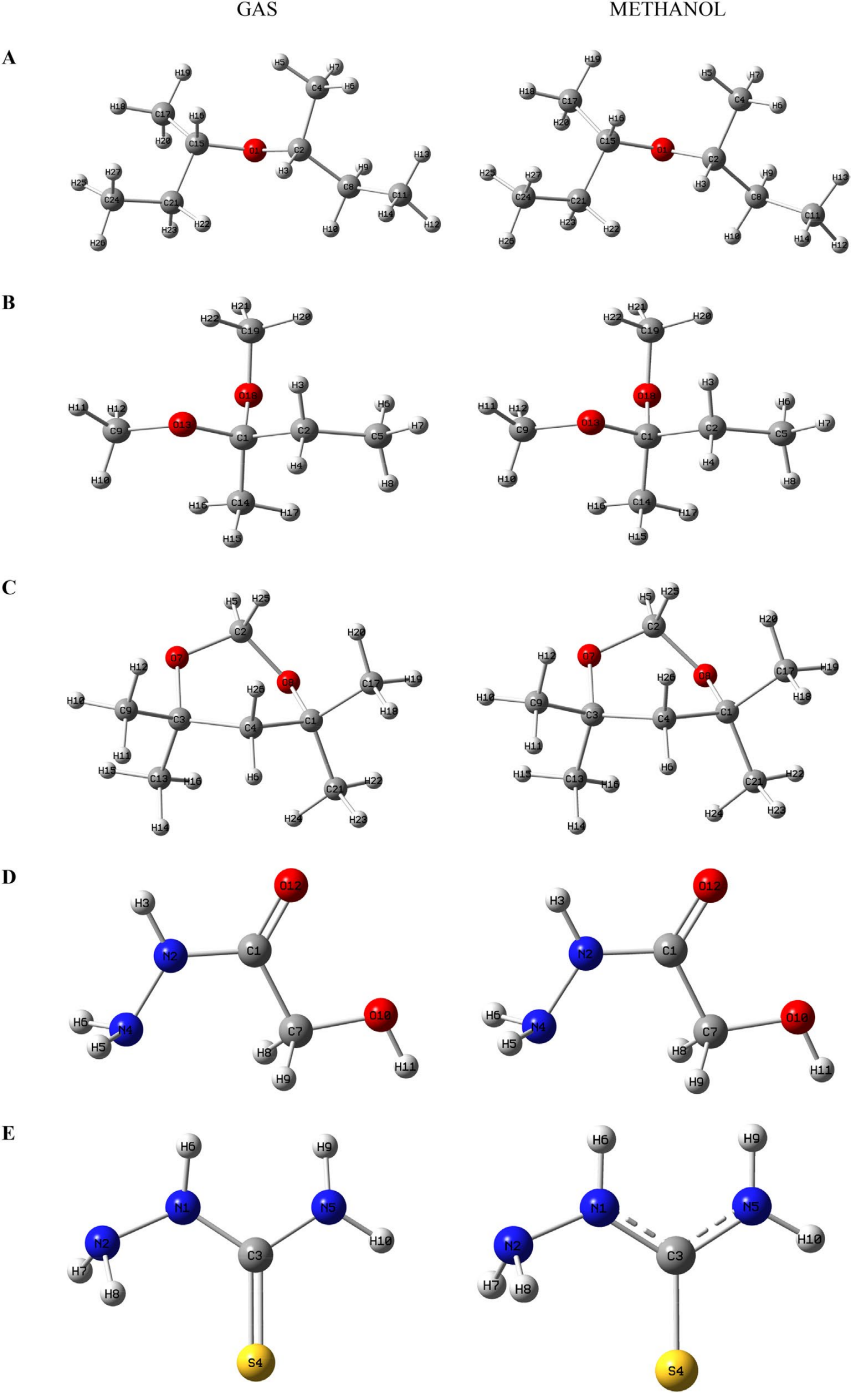
Optimized structures of (A) di‐sec‐butyl ether, (B) 2,2‐dimethoxybutane, (C) 4,4,6,6‐tetramethyl‐1,3‐dioxane, (D) hydroxyacetic acid, hydrazide, and (E) hydrazinecarbothioamide in gas phase and methanol phase.

**FIGURE 9:**
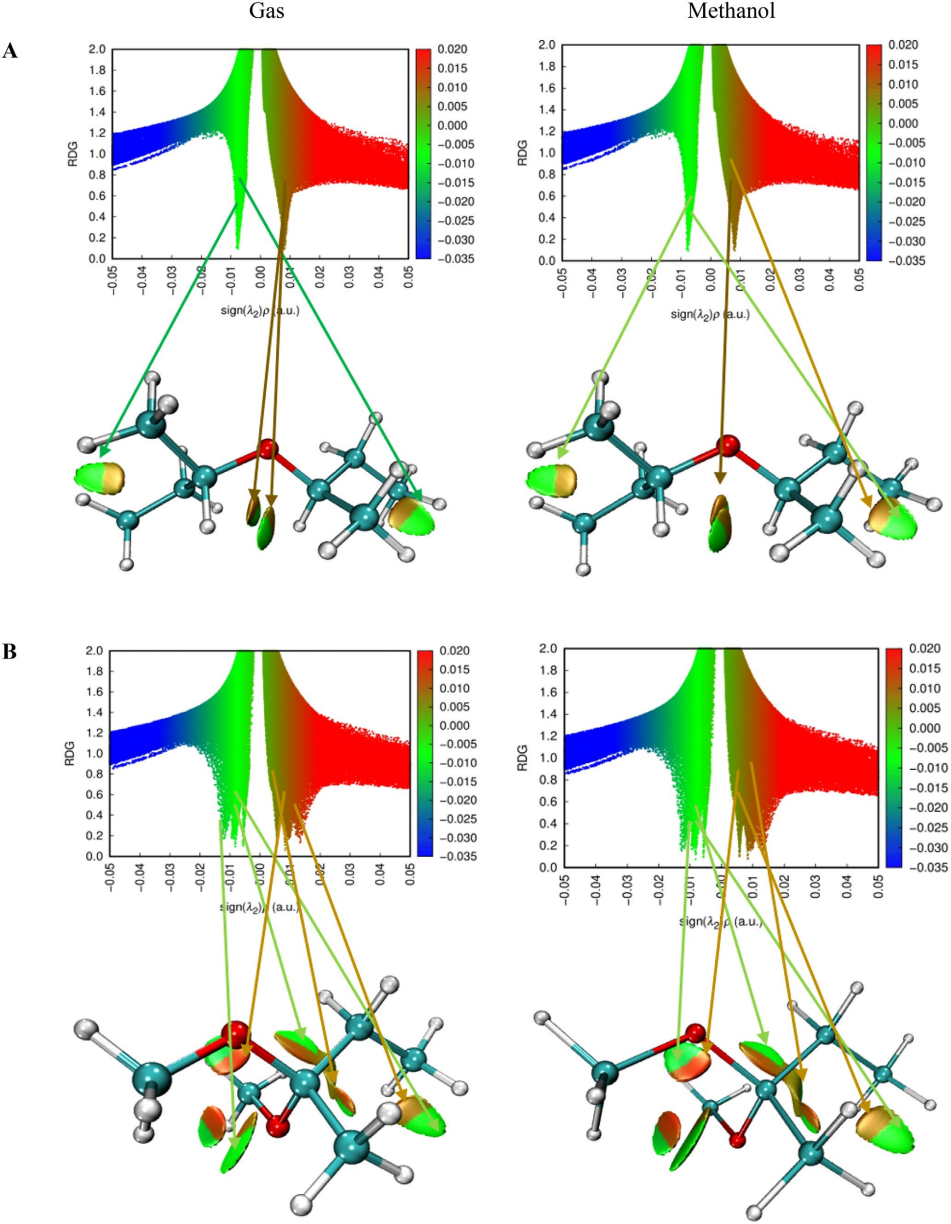
NCI–RDG plots of (A) di‐sec‐butyl ether and (B) 2,2‐dimethoxybutane in gas phase and methanol phase.

**FIGURE 10:**
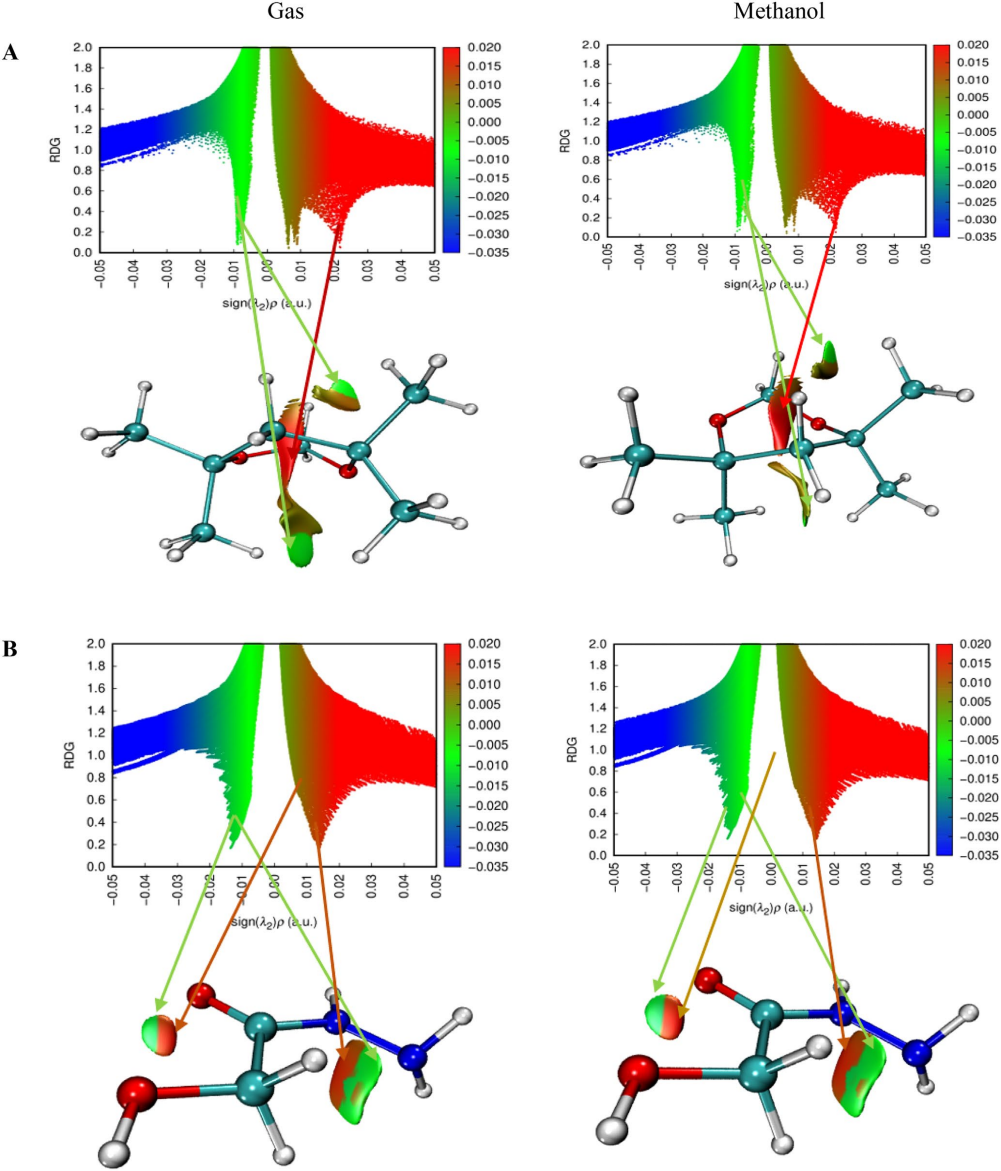
NCI–RDG plots of (A) 4,4,6,6‐tetramethyl‐1,3‐dioxane and (B) hydroxyacetic acid, hydrazide in gas phase and methanol phase.

**FIGURE 11:**
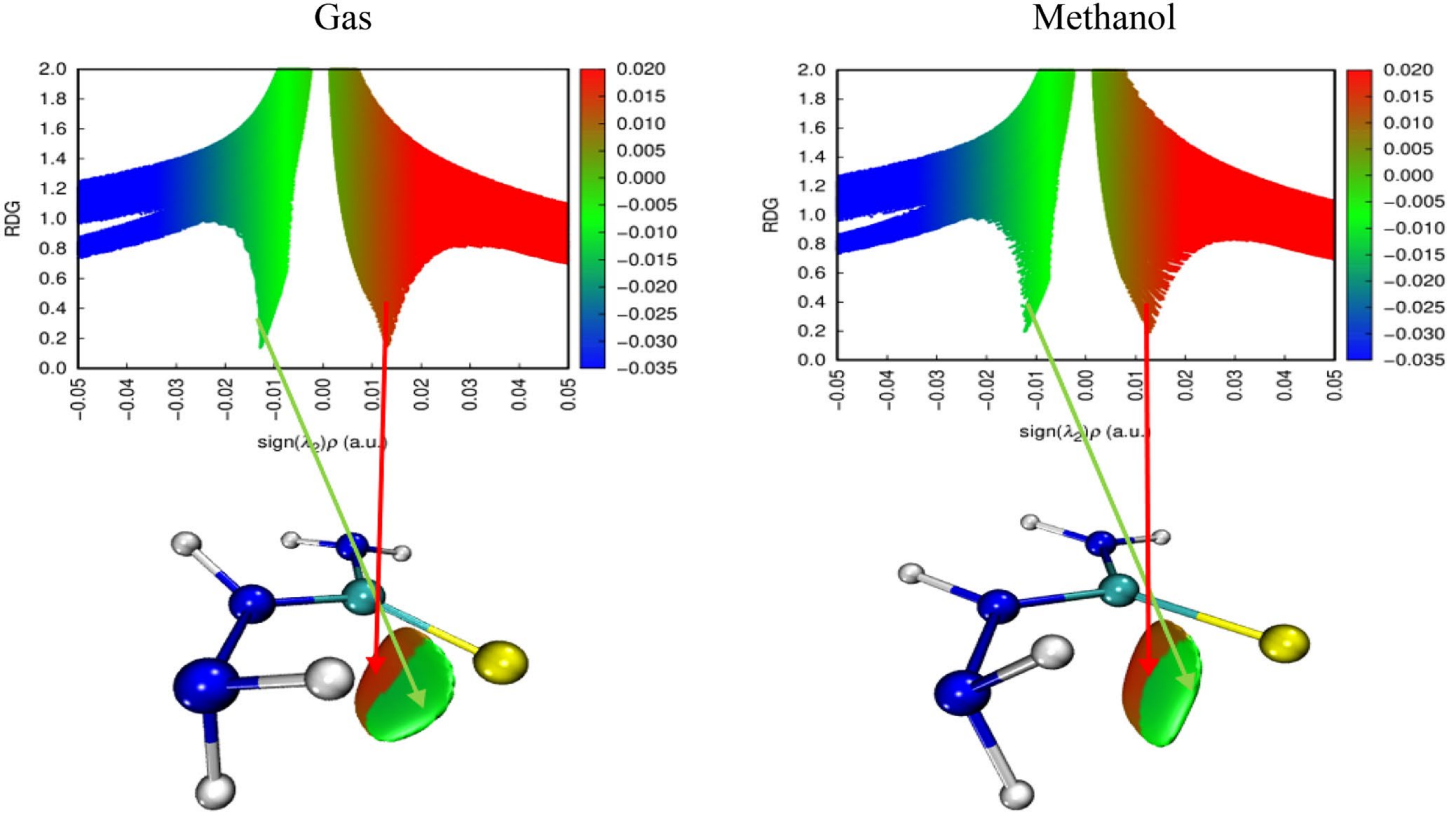
NCI–RDG plots of hydrazinecarbothioamide in gas phase and methanol phase.

**FIGURE 12:**
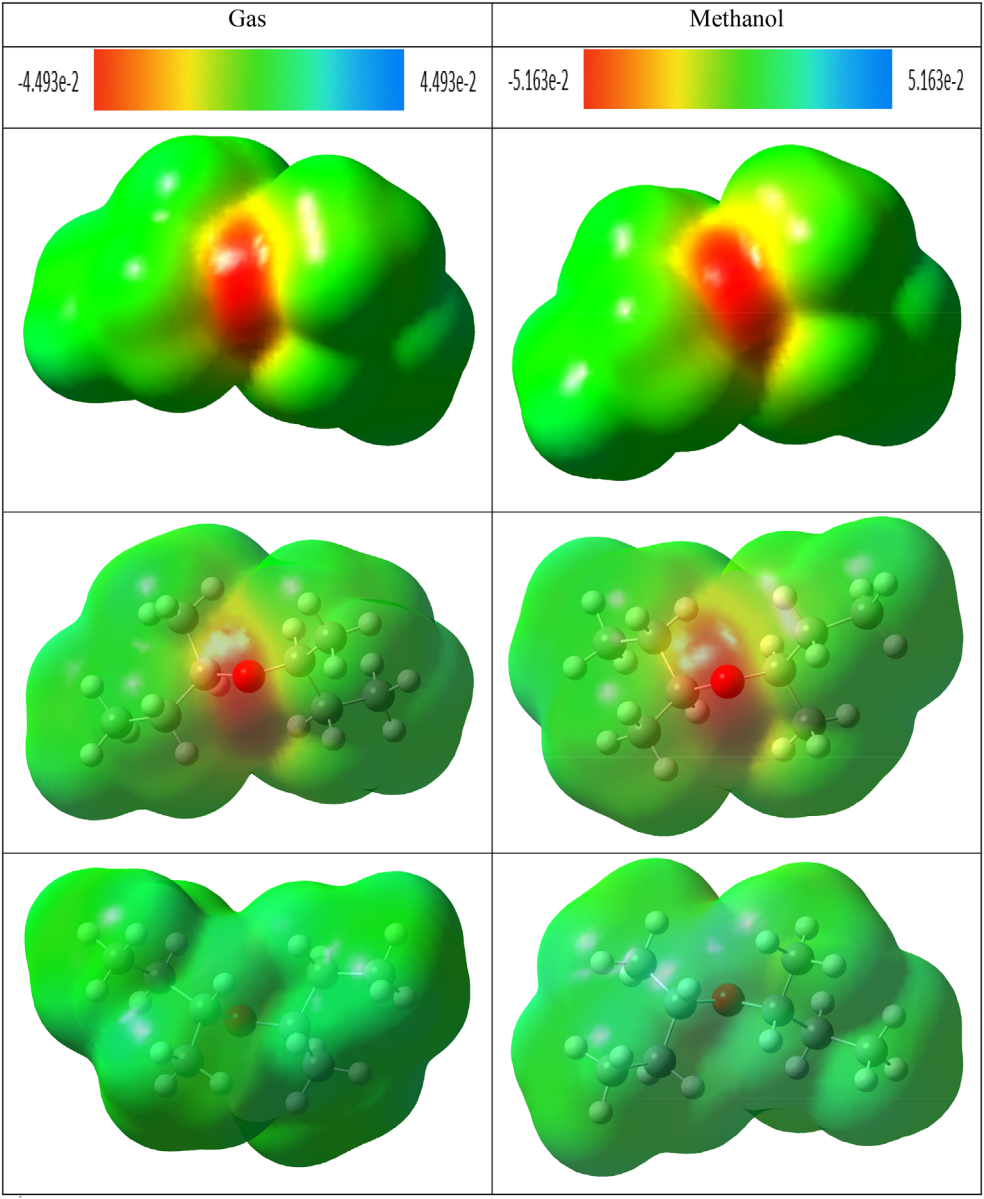
The MEP of di‐sec‐butyl ether in gas and methanol phase.

**FIGURE 13:**
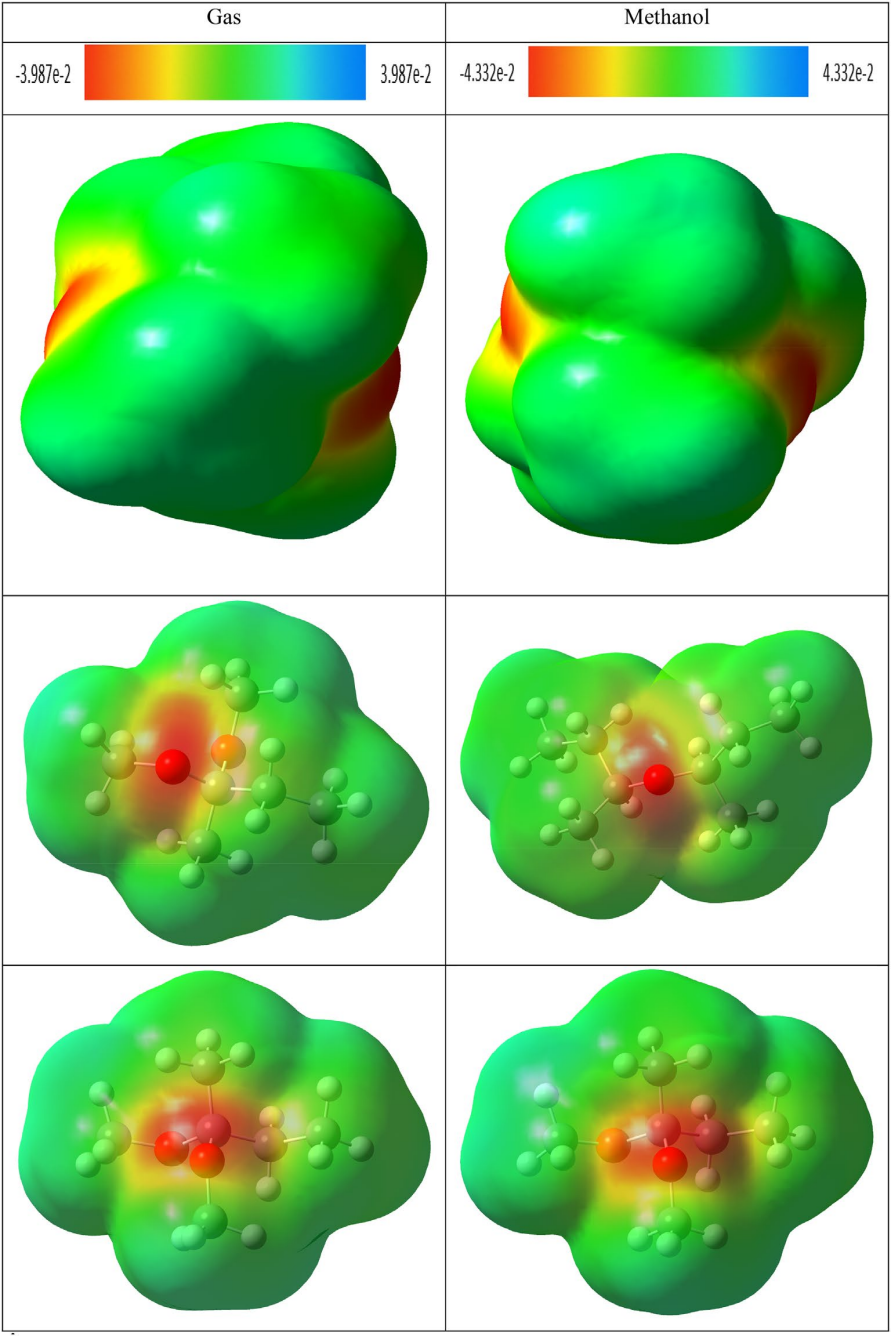
The MEP of 2,2‐dimethoxybutane in gas and methanol phase.

**FIGURE 14:**
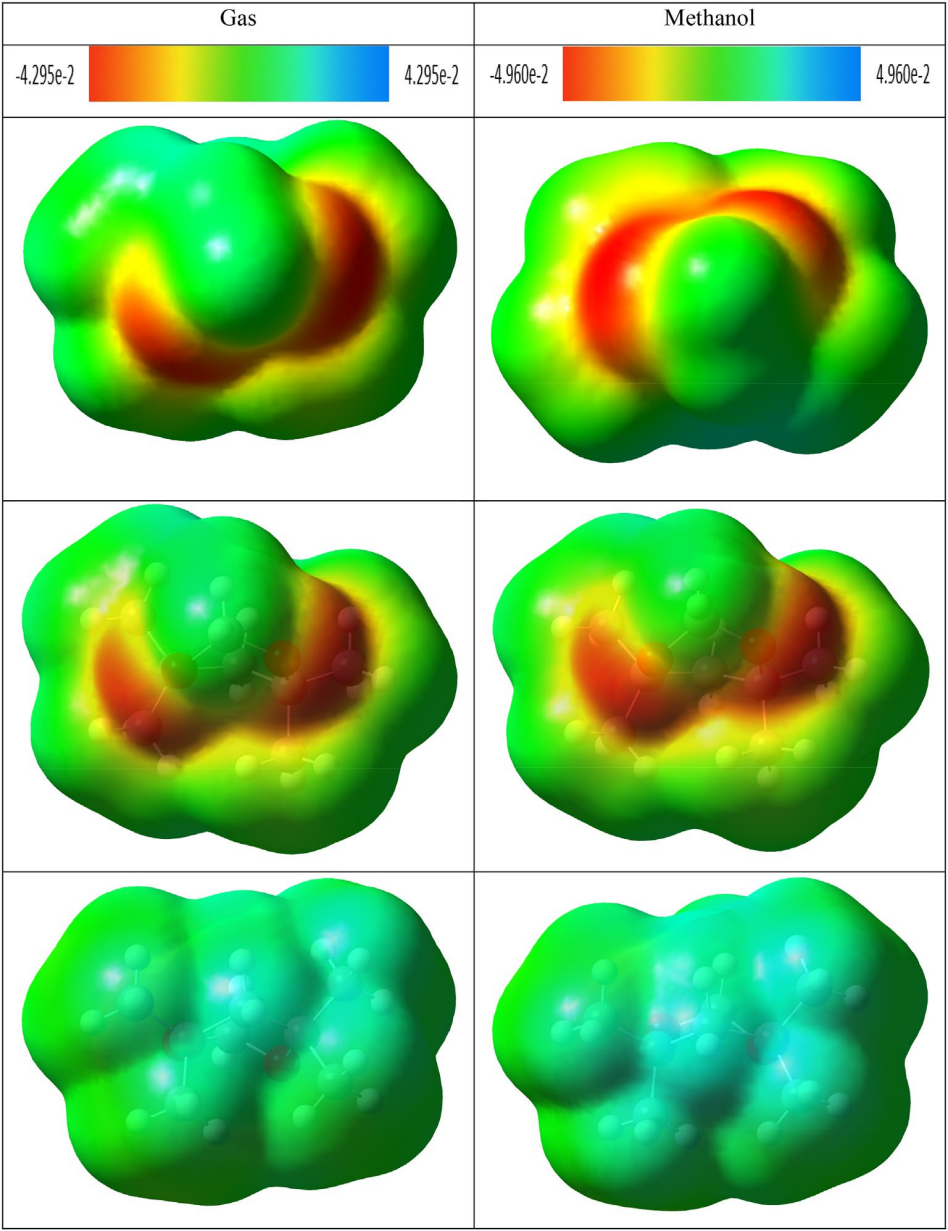
The MEP of 4,4,6,6‐tetramethyl‐1,3‐dioxane in gas and methanol phase.

**FIGURE 15:**
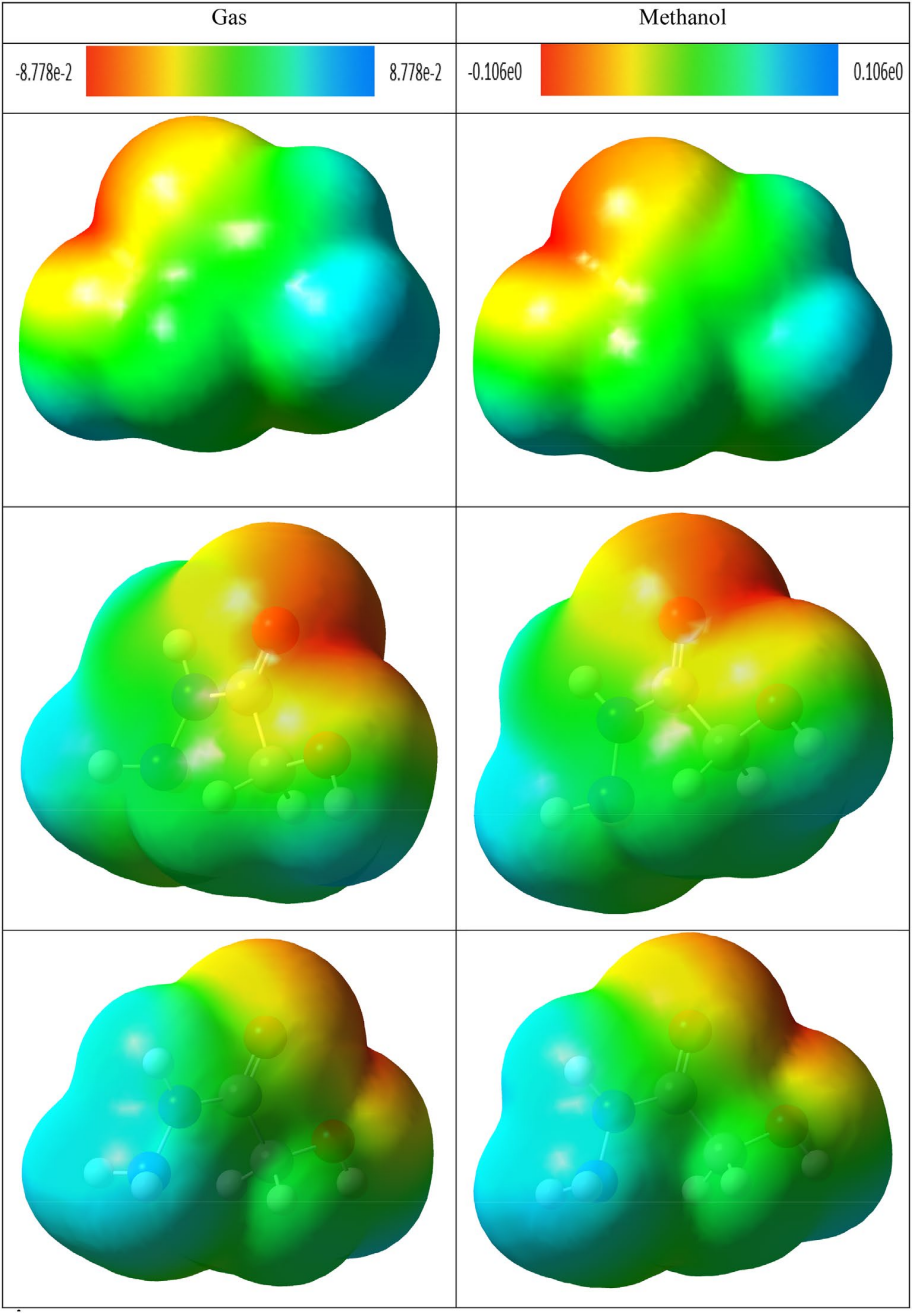
The MEP of hydroxyacetic acid, hydrazide in gas and methanol phase.

**FIGURE 16:**
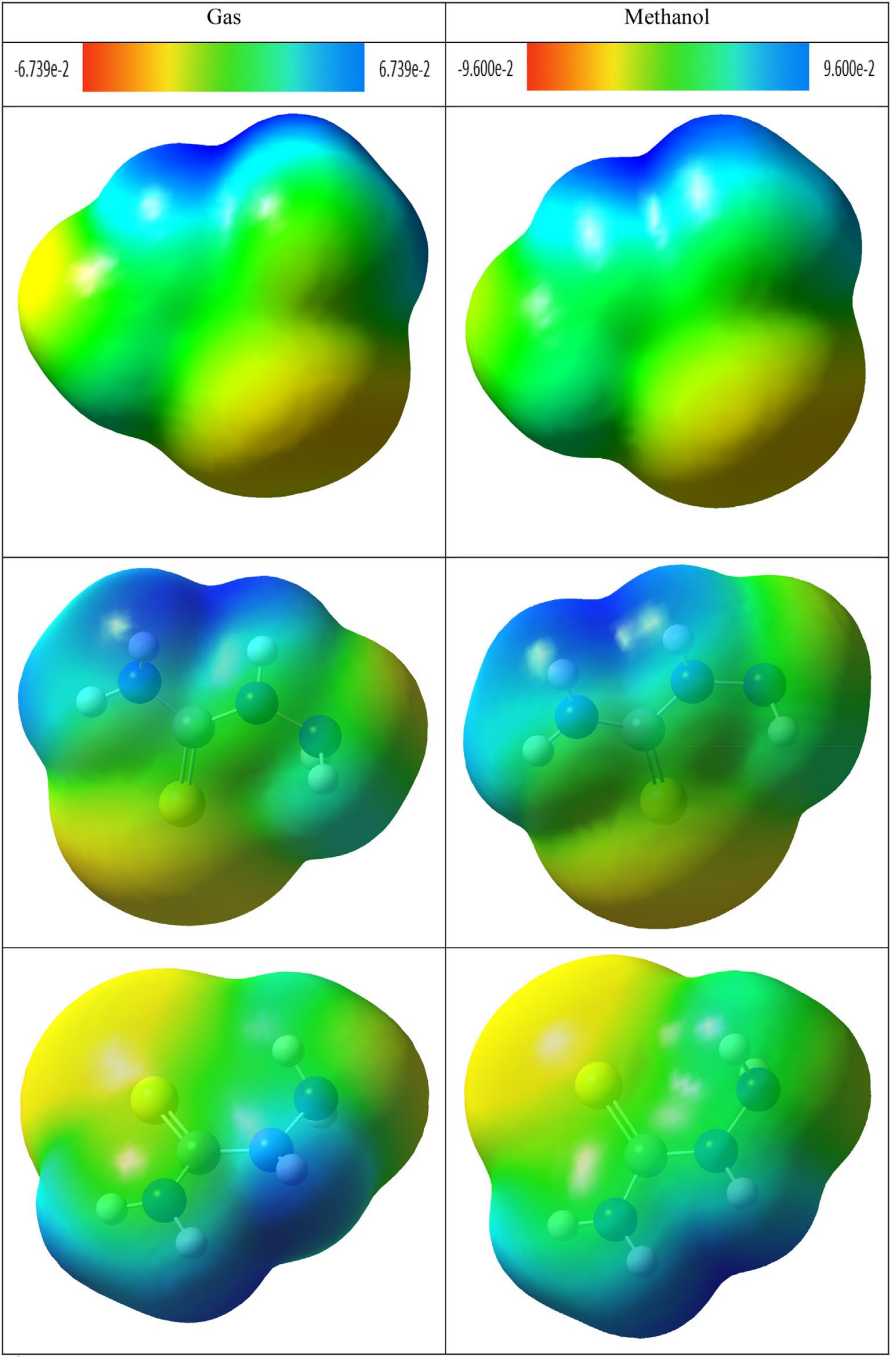
The MEP of hydrazinecarbothioamide in gas and methanol phase.

Molecular docking analysis revealed that phytochemicals identified from the *S. spiralis* seed extract exhibit varying affinities and interaction types with the selected protein target, as shown in Table 3. The compound 4,4,6,6‐tetramethyl‐1,3‐dioxane showed the highest binding affinity with a docking score of −6.3 kcal/mol, engaging in alkyl and Pi–alkyl interactions with residues such as LEU324, VAL4, ILE5, and LYS308 (Figure 3). Hydroxyacetic acid, hydrazide displayed a binding energy of −4.3 kcal/mol and formed multiple hydrogen bonds with TYR263, HIS72, and HIS73. Additionally, it was involved in attractive charge and Pi–cation interactions with ASP321 and HIS72 (Figure 4). Di‐sec‐butyl ether showed moderate binding affinity (−3.9 kcal/mol) and engaged in Pi–Pi stacking with TYR317, along with extensive alkyl and Pi–alkyl interactions with residues such as VAL61, VAL64, ILE80, PHE65, and PHE324 (Figure 5). Hydrazinecarbothioamide (−3.6 kcal/mol) formed hydrogen bonds with LYS1113, ALA1117, and LEU70 and showed a Pi–sulfur interaction with HIS72 (Figure 6). Similarly, 2,2‐dimethoxybutane, with the same binding score (−3.6 kcal/mol), interacted through Pi–alkyl contacts with HIS72, TYR317, and PHE324 (Figure 7). Overall, the findings suggest that 4,4,6,6‐tetramethyl‐1,3‐dioxane may act as a prominent ligand candidate due to its strong binding affinity and multiple hydrophobic interactions (Figures 8, 9, 10, 11, 12, 13, 14, 15, 16).

**TABLE 3:** Binding affinities and molecular interactions of selected *Spiranthes spiralis* seed compounds.

Compound	Binding scores (kcal/mol)	Hydrogen bond interactions	Pi–Pi stacking	Alkyl interactions
4,4,6,6‐Tetramethyl‐1,3‐dioxane	−6.3	—	—	LEU324–ligand VAL4–ligand Ligand–ILE5 (Pi–alkyl) C9–LYS308 (Pi–alkyl) C9–LEU324 (Pi–alkyl) C9–ILE5 (Pi–alkyl)
Hydroxyacetic acid, hydrazide	−4.3	TYR263:HH–O1, H1–HIS73:O, H2–HIS72:O, H2–HIS73:O, H3–HIS72:O, H4–HIS72:NE2	—	N1–ASP321: OD2 (attractive charge) N1–HIS72 (Pi–cation) N2–HIS72 (Pi–cation)
Di‐sec‐butyl ether	−3.9	—	H6–TYR317	C2–VAL64 C4–VAL61 C4–ILE80 C6–ILE80 PHE65–C2 (Pi–alkyl) PHE65–C4 (Pi–alkyl) TYR263–C6 (Pi–alkyl) TYR317–C6 (Pi–alkyl) TYR317–C8 (Pi–alkyl) PHE324–C2 (Pi–alkyl) PHE324–C4 (Pi–alkyl) PHE324–C8 (Pi–alkyl)
Hydrazinecarbothioamide	−3.6	H1–LYS1113: O H1–ALA1117: O H3–LEU70: O	—	S1–HIS72 (Pi–sulfur)
2,2‐Dimethoxybutane	−3.6	—	—	HIS72–C1 (Pi–alkyl) TYR317–C5 (Pi–alkyl) PHE324–C1 (Pi–alkyl) PHE324–C5 (Pi–alkyl)

Table 4 summarizes the ADMET and toxicity profiles of five compounds identified from *S. spiralis* seed extract. Hydroxyacetic acid, hydrazide, and hydrazinecarbothioamide tested positive for AMES toxicity, suggesting possible mutagenic potential, whereas the remaining compounds showed no such effect. Maximum tolerated doses ranged from 1.094 to 1.585 log mg/kg/day, indicating acceptable human safety margins. None of the compounds were predicted to inhibit hERG channels, reducing concerns of cardiotoxicity. LD_50_ values (mol/kg) suggested low acute toxicity, particularly for hydrazinecarbothioamide. However, this compound also showed the lowest LOAEL value (0.264 log mg/kg_bw/day), raising caution for chronic exposure. All compounds were non‐hepatotoxic, while 4,4,6,6‐tetramethyl‐1,3‐dioxane, di‐sec‐butyl ether, and 2,2‐dimethoxybutane showed potential for skin sensitization. Toxicity predictions for *Tetrahymena pyriformis* and minnow species varied, with hydroxyacetic acid and hydrazide being the most toxic in both models. These findings provide initial insights into the safety profiles of the investigated compounds for potential pharmacological or environmental applications.

**TABLE 4:** Predicted ADME properties and toxicity profile of selected compounds from *Spiranthes spiralis* seed extract.

Properties	4,4,6,6‐Tetramethyl‐1,3‐dioxane	Hydroxyacetic acid, hydrazide	Di‐sec‐butyl ether	Hydrazinecarbothioamide	2,2‐Dimethoxybutane
AMES toxicity	No	Yes	No	Yes	No
Max. tolerated dose (human) (log mg/kg/day)	1.094	1.585	1.402	1.576	1.399
hERG I inhibitor	No	No	No	No	No
hERG II inhibitor	No	No	No	No	No
Oral rat acute toxicity (LD_50_) (mol/kg)	2.302	2.376	2.239	2.647	2.164
Oral rat chronic toxicity (LOAEL) (log mg/kg_bw/day)	1.828	2.575	1.879	0.264	2.029
Hepatotoxicity	No	No	No	No	No
Skin sensitization	Yes	No	Yes	No	Yes
*Tetrahymena pyriformis* toxicity (log μg/L)	−0.492	−1.094	−0.205	−0.947	−0.857
Minnow toxicity (log mM)	2.054	3.862	1.312	3.523	1.884

### 3.6 Optimization Studies

Optimized structures of the chemical compounds shown in Figure 8 were obtained using DFT calculations at the B3LYP/6‐31G(d,p) level for both gas and methanol phases. The IEFPCM solvent model was used to model the solvent effect. In addition, vibrational frequency analysis was performed during geometry optimization for all compounds in both phases. The absence of negative or imaginary frequencies in all structures confirmed that the optimized structures corresponded to a real energy minimum, indicating that the obtained results were correct.

### 3.7 Frontier Molecular Orbital Analysis

Frontier molecular orbitals (FMOs), the HOMO and the LUMO, are critical in determining the electronic and reactive properties of a molecule (Vinduja et al. 2020). Although HOMO represents the electron donating capacity of a molecule and hence its nucleophilic character, LUMO indicates its electron receiving capacity and electrophilic character (Al‐Saadi 2020). The HOMO energy level (*E*_HOMO_) indicates the tendency of the molecule to act as an electron donor, while the LUMO energy level (*E*_LUMO_) refers to the capacity of the molecule to behave as an electron acceptor (Prabhu et al. 2025). A high *E*_HOMO_ value indicates an increasing tendency to give electrons, while a low *E*_LUMO_ value indicates an increasing tendency to receive electrons (Akram et al. 2019).

The HOMO–LUMO orbitals of the compounds are presented in Figures S1–S5. The orbital distributions of the gas and methanol phases of each compound are structurally similar, although they exhibit differences in terms of energy levels. This indicates that the solvent environment, that is, the solvation effect, plays a decisive role in determining the molecular orbital energies.

In di‐sec‐butyl ether, 2,2‐dimethoxybutane, 4,4,6,6‐tetramethyl‐1,3‐dioxane and hydroxyacetic acid hydrazide compounds, both HOMO and LUMO orbitals are homogeneously distributed throughout the molecular skeleton. In contrast, in hydrazinecarbothioamide, the distribution of these orbitals in space exhibits a more localized structure and is particularly concentrated on the C=S functional group of the compound. This indicates that the reactive sites of hydrazinecarbothioamide are more prominent, with electronic density concentrated in these sites.

### 3.8 Global Reactivity Parameters

The HOMO–LUMO energy gap (Δ*E*) is a fundamental parameter in understanding the chemical reactivity, stability, biological interaction potential and bond formation characteristics of molecules (Hasan et al. 2025). Calculation of these values allows global reactivity parameters to be obtained. These parameters include electron affinity (EA), ionization potential (IP), electronegativity (*χ*) (Mulliken 1934), chemical hardness (*η*) (Edwards and Pearson 1962), chemical potential (*μ*) (Parr and Pearson 1983), chemical softness (*ζ*) (Yang and Parr 1985), global electrophilicity index (*ω*) (Parr et al. 1999) and nucleophilicity index (*N*) (Edwards and Pearson 1962). These parameters are derived from the HOMO and LUMO energy levels based on Koopmans theorem (Koopmans 1934). It is assumed that the lower the Δ*E* values of molecules, the more chemically “soft” and reactive; the larger the Δ*E*, the molecule more “hard,” stable and less reactive (Abisha et al. 2023; Shukla et al. 2017). This has a direct determining effect on the biological activity potential of molecules. Global reactivity parameters are calculated according to the equations given below:

$$\text{Ionization potential}\;\left(\mathrm{IP}\right)=-E_{\text{HOMO}}$$

$$\text{Electron affinity}\;\left(\mathrm{EA}\right)=-E_{\text{LUMO}}$$

$$\Delta E_{\mathrm{gap}}\;\left(\text{energy}\;\mathrm{gap}\right)=E_{\text{LUMO}}-E_{\text{HOMO}}$$

$$\text{Electronegativity}\;\left(\chi \right)=\left(\mathrm{IP}+\mathrm{EA}\right)/2$$

$$\text{Chemical potential}\;\left(\mu \right)=-\left(\mathrm{IP}+\mathrm{EA}\right)/2$$

$$\text{Chemical hardness}\;\left(\eta \right)=\left(\mathrm{IP}-\mathrm{EA}\right)/2$$

$$\text{Global softness}\;\left(\zeta \right)=1/\left(2\eta \right)$$

$$\text{Global electrophilicity index}\;\left(\omega \right)=\mu^{2}/2\eta$$

$$\text{Nucleophilicity index}\;\left(N\right)=1/\omega$$

The HOMO–LUMO orbital energy levels of the compounds and the calculated global reactivity parameters are summarized in Table S1. According to the *E*_gap_ values obtained, the general ranking is as follows: 2,2‐Dimethoxybutane (methanol) > 2,2‐dimethoxybutane (gas) > di‐sec‐butyl ether (methanol) > 4,4,6,6‐tetramethyl‐1,3‐dioxane (methanol) > di‐sec‐butyl ether (gas) > 4,4,6, 6‐tetramethyl‐1,3‐dioxane (gas) > hydroxyacetic acid, hydrazide (methanol) > hydroxyacetic acid, hydrazide (gas) > hydrazinecarbothioamide (gas) > hydrazinecarbothioamide (methanol).

As can be seen from this ranking, except for hydrazinecarbothioamide, the *E*_gap_ values of all other compounds in the methanol phase were slightly higher than in the gas phase. This indicates that the chemical reactivities of these compounds do not show a significant change in the solvent medium, thus methanol has a limited solvation effect on these molecules. On the other hand, the opposite trend was observed for hydrazinecarbothioamide, with the *E*_gap_ value in the methanol phase being lower than in the gas phase. This result reveals that it may have a more active structure both chemically and biologically in a methanol medium, and solvation is effective in increasing reactivity.

In this study, analyses were carried out only in the methanol phase. Accordingly, the reactivity ranking of the compounds according to *E*_gap_ values in methanol medium is as follows: 2,2‐Dimethoxybutane > di‐sec‐butyl ether > 4,4,6,6‐tetramethyl‐1,3‐dioxane > hydroxyacetic acid, hydrazide > hydrazinecarbothioamide. 2,2‐dimethoxybutane, with the highest *E*_gap_ value (9.390 eV), is the most electronically stable and chemically inert compound. In contrast, the hydrazinecarbothioamide compound has the lowest *E*_gap_ value (3.423 eV), suggesting high chemical reactivity and potential biological activity due to the narrow energy difference between the boundary molecular orbitals.

Global reactivity parameter evaluations also support this trend. Hydrazinecarbothioamide exhibited the lowest chemical hardness (*η* = 1.711 eV) and the highest chemical softness (*ζ* = 0.292 1/eV, ζ = 1/η) values, indicating high chemical flexibility and potential to interact with biological targets. On the other hand, the other compounds close to each other with high *η* and *ζ* values reveal that they tend to be resistant to chemical deformation and low biological reactivity compared to the hydrazinecarbothioamide compound.

The electrophilicity (*ω*) and nucleophilicity (*N*) values show that the hydrazinecarbothioamide compound has the highest *ω* value with 4.453 eV and the lowest *N* value with 0.224, showing a strong electron acceptor character and high interaction potential in nucleophilic environments. The electronegativity (*χ*) and chemical potential (*μ*) values are also consistent with these results. The highest electronegativity (*χ* = 3.904 eV) and the most negative chemical potential (*μ* = −3.904 eV) values obtained in the hydrazinecarbothioamide compound, respectively, indicate that this molecule has a strong tendency to attract electrons from the external environment and exhibits a distinct electrophilic character. In particular, the negative chemical potential supports the high instability level and increased reactivity potential of the molecule, indicating that it may have a high capacity to interact with biological systems. Other compounds exhibit a more stable electronic structure with lower *χ*, *ω*, high *N*, and less negative *μ* values.

The ionization potential (IP) shows that the 2,2‐dimethoxybutane compound has the highest IP value (7.104 eV), which supports its low reactivity. Hydrazinecarbothioamide, on the other hand, has a low IP value of only 5.616 eV, pointing out that it may lose electrons more easily and have a high binding capacity.

The spatial distributions of HOMO and LUMO orbitals also support these chemical behaviors. In the hydrazinecarbothioamide compound, both HOMO and LUMO orbitals are localized mainly on the C=S group, indicating that the reactive sites of the compound are concentrated and have high interaction potential. In other compounds (e.g., di‐sec‐butyl ether, 2,2‐dimethoxybutane), these orbitals were more widely distributed throughout the molecular skeleton, favoring a low reactivity trend.

As a result, among the compounds studied, hydrazinecarbothioamide was evaluated as the compound with the highest chemical reactivity and potential biological interaction capacity, standing out with its narrow *E*_gap_ gap, high electrophilicity value, and HOMO–LUMO orbitals localized to the C=S group. On the other hand, 2,2‐dimethoxybutane, 4,4,6,6‐tetramethyl‐1,3‐dioxane, di‐sec‐butyl ether, and hydroxyacetic acid, hydrazide compounds are chemically stable, low reactive, and biologically behave inert structures in methanol medium, exhibiting high *E*_gap_ and chemical hardness (*η*) values and low electrophilicity values (*ω*). While these four compounds can be evaluated as carrier or supporting molecules in possible pharmacological systems, it is predicted that hydrazinecarbothioamide may assume a decisive role in the formation of pharmacological or antioxidant effects in the systems in which it is involved.

### 3.9 Non‐Covalent Interaction (NCI) and Reduced Density Gradient (RDG) Analyses

Non‐covalent interactions are defined as weak forces between different regions of a molecule or between different molecules (Cesario 2019). These interactions play a significant role in the structural stability, chemical reactivity, and biological functionality of molecules (Hajji et al. 2021). The main examples of this phenomenon include hydrogen bonds, van der Waals forces, and steric repulsions (Kose 2021).

One of the most effective computational approaches used to determine these interactions at the molecular level is RDG analysis (Saleh et al. 2015). The RDG–NCI analysis is a computational method that identifies both intramolecular and intermolecular weak interaction regions in molecular systems. (Medimagh et al. 2021) This analysis is based on the electron density and the rate of change of this density in space (Cukrowski et al. 2015). The RDG value is a dimensionless parameter, which is defined as follows (Silvi and Savin 1994):

$$\mathrm{RDG}\left(r\right)=\frac{1}{2{\left(3\pi^{2}\right)}^{1/3}}\frac{|\nabla \rho \left(r\right)|}{\;\rho {\left(r\right)}^{4/3}}$$

$\nabla \rho \left(r\right)$ is the gradient of electron density in space. *ρ* is the electron density at a given point.

The classification of non‐covalent interactions is determined by the relationship between the second eigenvalue of the Hessian matrix (*λ*_2_) and the electron density (*ρ*) (Shi et al. 2021).

According to this classification:

- Attractive interactions (*λ*_2_ < 0 and *ρ* > 0): Represent hydrogen bonds, halogen bonds, and dipole–dipole interactions and are indicated by the blue color.
- Weak van der Waals interactions (*λ*_2_ ≈ 0 and *ρ* ≈ 0): Describe regions of low electron density and are colored green.
- Repulsive/steric interactions (*λ*_2_ > 0 and *ρ* > 0): Represent steric repulsions and are indicated by the red color (Benyahlou et al. 2023; Ghalla et al. 2018).

These interactions are presented in two‐dimensional graphs and three‐dimensional isosurface visualizations to analyze in detail both the location and nature of interactions on the molecular surface. NCI–RDG analysis is a widely utilized technique in the field of molecular biology, particularly in the context of elucidating the structural‐functional relationships of large molecular systems and in molecular design studies (Saleh et al. 2015).

NCI–RDG graphs and isosurface images of the components analyzed in this study are presented in Figure S6. As a result of analyzing the obtained figures, it was observed that the NCI–RDG surfaces of both the gas and methanol phases of each compound were very similar. Weak van der Waals interactions expressed by light and dark green regions were detected in all compounds.

In the compound 4,4,6,6,6‐tetramethyl‐1,3‐dioxane, which has a ring structure, steric repulsion interactions are evident with the green colored region and are expressed by dark red colored regions. In the methanol phase, steric repulsions in this compound were observed to decrease. However, it is understood that the green colored regions at the top and bottom of the ring structure indicate van der Waals interaction potential. This can be interpreted as weak interactions with target molecules through the ring structure of the compound in question. The most remarkable finding in the NCI–RDG analysis was observed in hydroxyacetic acid, hydrazide and especially hydrazinecarbothioamide compounds (Figures 9, 10, 11). Although in these compounds no distinct “spike” (pointed structure) was observed in the blue regions known to correspond to hydrogen bonds, the presence of slender blue bands indicates that strong intermolecular interactions, especially hydrogen bonds, are present in these molecules (Liu et al. 2022).

### 3.10 Molecular Electrostatic Potential (MEP) Analysis

MEP is a fundamental quantum chemical analysis method that allows quantitative and visual examination of the spatial distribution of the electrostatic field generated by electrons and nuclei in a molecule (Ben Issa et al. 2020). This analysis reveals the site of chemical reactivity by evaluating the electrostatic potential densities on the surface of the molecule and plays a critical role in determining the electrophilic and nucleophilic interaction sites (Politzer and Murray 2021).

MEP maps are based on the three‐dimensional visualization of electrostatic potential values calculated on the surface of molecules through color coding (Hussein et al. 2022). Generally, red colors define electron‐rich regions with negative electrostatic potentials, which are favorable for electrophilic attacks. In contrast, blue colors indicate electron‐poor areas with positive potential and represent regions favored for nucleophilic attacks. Green and yellow tones generally correspond to neutral or weak interaction regions (Gebremeskel et al. 2024; Kansız et al. 2022).

MEP analysis is an effective method not only for the prediction of molecular reactivity but also for the evaluation of hydrogen bonding, proton affinity, charge transfer, and intermolecular interactions (Kenny 2009; Suresh et al. 2022). It is also widely used in drug design and structure–activity relationship (SAR) studies to evaluate the electrostatic compatibility of ligand‐receptor interactions (Scrocco and Tomasi 2007). Considering this, MEP surfaces provide valuable information both qualitatively and quantitatively in predicting selectivity and binding properties at the molecular level (Kosar and Albayrak 2011).

The potential distributions of the MEP maps of di‐sec‐butyl ether (Figure 12), 2,2‐dimethoxybutane (Figure 13) and 4,4,6,6‐tetramethyl‐1,3‐dioxane (Figure 14) in the methanol phase did not show a significant change compared to their gas phase. On the other hand, more prominent changes were observed in the electrostatic potential surfaces of hydroxyacetic acid, hydrazide, and especially hydrazinecarbothioamide compounds in the methanol phase.

The MEP of di‐sec‐butyl ether, 2,2‐dimethoxybutane, and 4,4,6,6‐tetramethyl‐1,3‐dioxane compounds show structural similarity. In these compounds, the surroundings of oxygen atoms in the molecular structure are represented by red color as electron‐rich regions. The remaining skeletal structure of the molecules is generally shown in green tones, suggesting that these regions are neutral or weakly interacting areas in terms of electrostatic potential.

In the MEP of hydroxyacetic acid, hydrazide compound (Figure 15), red colors were observed in the regions where O–H and C=O groups are present in the structure. Especially the dark red regions seen around the C=O group reveal that this region is one of the most electron‐rich regions. In addition, blue color was observed on hydrogen atoms in the N–H and –NH_2_ groups in the compound and hydrogens connected to the O–H group. This indicates that these regions are electron‐poor and can serve as potential hydrogen bond donor centers.

In hydrazinecarbothioamide compound, obvious blue colored regions were observed on hydrogen atoms in –NH_2_ and –NH groups connected to C–S group. This shows that these regions are electrophilic and can form hydrogen bonds. In addition, bright orange regions around the sulfur (S) atom are observed and this area has a moderate electrostatic density. The rest of the molecule is represented by green and yellow shades, indicating the presence of regions with more neutral potentials (Figure 16).

NCI–RDG and MEP analyses of di‐sec‐butyl ether compound agreed with molecular docking results. The di‐sec‐butyl ether compound formed only alkyl and Pi–alkyl hydrophobic interactions with the 6LI1 protein without hydrogen bonding or electrostatic interactions. This agrees with the electron‐neutral (green) regions observed in MEP analyses and the weak van der Waals interactions detected on NCI–RDG surfaces.

NCI–RDG and MEP analyses of 2,2‐dimethoxybutane compound agreed with molecular docking results. This compound formed only Pi–alkyl and van der Waals characterized hydrophobic interactions with the 6LI1 protein; it did not show hydrogen bonding or electrostatic interaction. These bond types are consistent with the electron‐neutral (green) regions observed in MEP analyses and the weak van der Waals interactions expressed by the light green areas detected on NCI–RDG surfaces.

4,4,4,6,6‐tetramethyl‐1,3‐dioxane was found to form only hydrophobic bonds of alkyl, Pi–alkyl, and van der Waals type in molecular docking interactions with 6LI1 protein. This binding profile is consistent with the fact that most of the molecular surface is represented by green hues in the MEP map, which are electrostatically neutral, low interaction potential regions. Similarly, the green regions observed in the NCI–RDG surface analyses indicate weak van der Waals interactions dominating the overall molecule. When these findings are evaluated together, it is concluded that the compound binds to the protein with hydrophobic and surface‐based weak bonds, and there is a high level of agreement between the three analysis methods.

The docking, MEP, and NCI–RDG analyses of hydroxyacetic acid, hydrazide compound show a high level of agreement with each other. In the docking analysis, this compound formed many classical hydrogen bonds with amino acids such as ASP321, HIS72, HIS73, and TYR263 in the 6LI1 protein; it also showed electrostatic interactions of Pi–cation and attractive charge type. This binding profile corresponds directly to the dark red regions (electron‐rich centres) observed in the MEP map, particularly around the C=O and O–H groups, as well as the prominent blue color distribution on hydrogen atoms in the N–H and NH_2_ groups (hydrogen bond donor regions). In the NCI–RDG analysis, the presence of broad blue bands indicates the presence of strong intermolecular hydrogen bonds in this compound, although classical pointed “spike” structures are not observed. There are also weak van der Waals interactions on the remaining surface of the molecule, as indicated by the light and dark green regions. In this context, when the three analysis methods are evaluated together, it is understood that the hydroxyacetic acid, hydrazide compound exhibits a stable and specific interaction profile with the target protein with both electrostatic and directional hydrogen bonds.

The docking, MEP, and NCI–RDG analyses of the hydrazinecarbothioamide compound show consistent and supportive results. In the docking analysis, this compound established classical hydrogen bonds and one Pi–sulfur interaction with the 6LI1 protein through amino acid residues LEU70, ALA1117, LYS1113, and HIS72. These bond types indicate that the molecule is prone to strong and directional intermolecular interactions. The MEP analysis revealed positive regions on the hydrogen bonding groups, in agreement with the broad blue bands observed in the NCI–RDG analysis; thus, both analyses confirmed that the molecule carries hydrogen bond donor centres. Furthermore, the light and dark green regions on the surface of the molecule represent both the electrostatic neutral sites in MEP and the weak van der Waals interactions in NCI–RDG analysis, complementing the overall bonding profile of the molecule. In this context, the hydrazinecarbothioamide compound binds to the protein both by selective interactions based on hydrogen bonds and by weak surface contacts, showing a strong agreement between the three analysis methods.

HOMO–LUMO energy levels and global reactivity parameters comprehensively supported the results obtained from MEP, NCI–RDG and molecular docking analyses. In particular, the hydrazinecarbothioamide compound exhibited high chemical reactivity and electrophilic character with the lowest *E*_gap_ value and the highest electrophilicity index (*ω*); this feature appeared in agreement with the high ligand efficiency and low inhibitor concentration (Ki) of the molecule in the docking analysis. In addition, the presence of blue coloration at hydrogen bond donor sites in MEP maps and broad blue bands observed in the absence of classical spikes in NCI–RDG analysis supported the potential for strong directional intermolecular interactions. On the other hand, 2,2‐dimethoxybutane and 4,4,6,6‐tetramethyl‐1,3‐dioxane compounds showed low reactivity with higher *E*_gap_ and chemical hardness (*η*) values; this is in agreement with the fact that these compounds present a binding profile limited to dominant neutral (green) areas on MEP surfaces and only weak van der Waals interactions in NCI–RDG analyses. Furthermore, the ADMET data provided a significant contribution to the evaluation of the biological suitability of these molecules; hydrazinecarbothioamide, despite its high binding capacity, carries toxicological risks such as skin sensitivity and low LD_50_, while 2,2‐dimethoxybutane exhibited a more balanced pharmaceutical profile with its low toxicity, high absorption and hepatotoxicity‐free structure. When all these data are evaluated together, it is understood that the results obtained in terms of electronic, surface and biological properties are consistent with each other and integrity between the analyses is provided.

## 4 Conclusion

The seeds of *S. spiralis* are characterized by a distinct fusiform morphology and structural uniformity along their anticlinal walls. FTIR analysis confirmed the presence of functional groups associated with cellulose, hemicellulose, lignin, and proteins. High total phenolic and proanthocyanidin contents in the methanolic extract were correlated with strong antioxidant activity, as evidenced by DPPH radical scavenging assays. GC–MS profiling revealed bioactive compounds such as 2,2‐dimethoxybutane and hydrazinecarbothioamide, whose molecular docking, MEP, and NCI–RDG analyses demonstrated significant binding affinity and directional hydrogen bonding interactions with the target protein. HOMO–LUMO and global reactivity parameters indicated that hydrazinecarbothioamide exhibits the highest chemical reactivity and biological interaction potential, whereas the other compounds display lower reactivity and higher chemical stability. Collectively, these findings highlight the seeds of *S. spiralis* as a rich source of phenolic compounds with potent antioxidant properties, while also identifying specific bioactive constituents with pharmacological potential, underscoring the importance of further investigation of terrestrial orchids as promising candidates for natural product‐based drug and functional food development.

## Author Contributions

**Erdi Can Aytar:** conceptualization, methodology, investigation, writing – original draft, project administration, data curation, supervision, visualization. **Taşkın Basılı:** investigation, data curation, resources, writing – review and editing. **Altevir Rossato Viana:** investigation. **Bengisu Şentürk:** investigation, data curation. **Emine İncilay Torunoğlu:** writing – original draft, visualization. **Major Mabuza:** writing – review and editing. **Mika Sillanpää:** methodology, resources, writing – review and editing, validation. **Yasemin Özdener Kömpe:** writing – review and editing.

## Funding

The authors have nothing to report.

## Ethics Statement

The authors have nothing to report.

## Conflicts of Interest

The authors declare no conflicts of interest.

## Supporting information


**Figure S1:** HOMO–LUMO orbitals of di‐sec‐butyl ether in gas phase and methanol phase.
**Figure S2:** HOMO–LUMO orbitals of 2,2‐dimethoxybutane in gas phase and methanol phase.
**Figure S3:** HOMO–LUMO orbitals of 4,4,6,6‐tetramethyl‐1,3‐dioxane in gas phase and methanol phase.
**Figure S4:** HOMO–LUMO orbitals of hydroxyacetic acid, hydrazide in gas phase and methanol phase.
**Figure S5:** HOMO–LUMO orbitals of hydrazinecarbothioamide in gas phase and methanol phase.
**Figure S6:** Classification of NCI using RDG isosurfaces with *λ*_2_–*ρ* values and color representation.
**Table S1:** Calculated HOMO–LUMO orbital energies, energy gaps (*E*_gap_), and global reactivity parameters of the compounds.

## Data Availability

The data that support the findings of this study are available from the corresponding author upon reasonable request.
