# Supplementary material for: Chemical Fingerprinting of Spiranthes spiralis L. Methanol Seed Extract: Spectroscopic, Chromatographic, and Computational Approaches
Source: Food Sci Nutr. 2026 Feb 11;14(2):e71531. doi: 10.1002/fsn3.71531 (PMC12896393; doi:10.1002/fsn3.71531)
Supplement: Supplementary file 1 — Figure S1: HOMO–LUMO orbitals of di‐sec‐butyl ether in gas phase and methanol phase. Figure S2: HOMO–LUMO orbitals of 2,2‐dimethoxybutane in gas phase and methanol phase. Figure S3: HOMO–LUMO orbitals of 4,4,6,6‐tetramethyl‐1,3‐dioxane in gas phase and methanol phase. Figure S4: HOMO–LUMO orbitals of hydroxyacetic acid, hydrazide in gas phase and methanol phase. Figure S5: HOMO–LUMO orbitals of hydrazinecarbothioamide in gas phase and methanol phase. Figure S6: Classification of NCI using RDG isosurfaces with λ 2–ρ values and color representation. Table S1: Calculated HOMO–LUMO orbital energies, energy gaps (E gap), and global reactivity parameters of the compounds. [file FSN3-14-e71531-s001.docx]

**Chemical Fingerprinting of *Spiranthes spiralis* L. Methanol Seed Extract: Spectroscopic, Chromatographic, and Computational Approaches**

**Erdi Can Aytar^1^*, Taşkın Basılı ^2^, Altevir Rossato Viana^3^, Bengisu Şentürk^4^, Emine İncilay Torunoğlu^5^,** **Major Mabuza^7^, Mika Sillanpää^6,7,8,9^, Yasemin Özdener Kömpe^4^**

^1^Usak University, Faculty of Agriculture, Department of Horticulture, Uşak, 64200, Türkiye

^2^ Ondokuz Mayıs University, Faculty of Science, Department of Chemistry, 55139, Samsun, Türkiye

^3^Federal University of Santa Maria, Department of Biochemistry and Molecular Biology, Santa Maria, Brazil.

^4^ Ondokuz Mayıs University, Faculty of Science, Department of Biology, Samsun, 55100, Türkiye

^5^ Necmettin Erbakan University, Faculty of Medicine, Department of Medical Biochemistry, Konya, Türkiye

^6^ Key Laboratory of Northwest Water Resource, Environment and Ecology, MOE, Xi’an University of Architecture and Technology, Xi’an 710055, China

^7^ Department of Chemical Engineering Technology, Faculty of Engineering and the Built Environment, University of Johannesburg, Johannesburg 2088, Republic of South Africa

^8^ Institute for Nanotechnology and Water Sustainability (iNanoWS), Florida Campus, College of Science, Engineering and Technology, University of South Africa, Johannesburg, 1710, South Africa

^9^ Centre of Research Impact and Outcome, Chitkara University Institute of Engineering and Technology, Chitkara University, Rajpura-140401, Punjab, India

|  | Gas | Methanol |
| --- | --- | --- |
| HOMO | 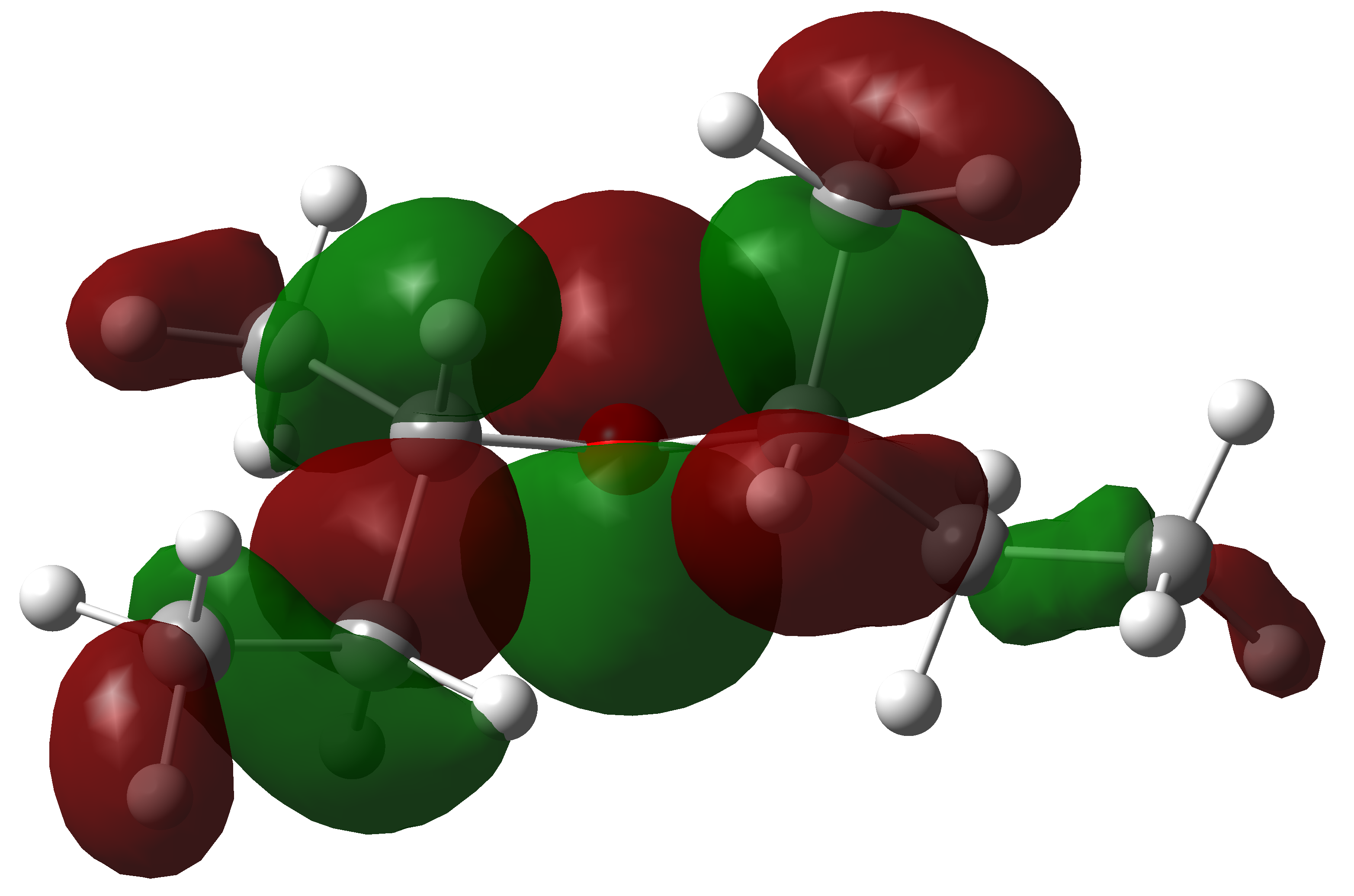 | 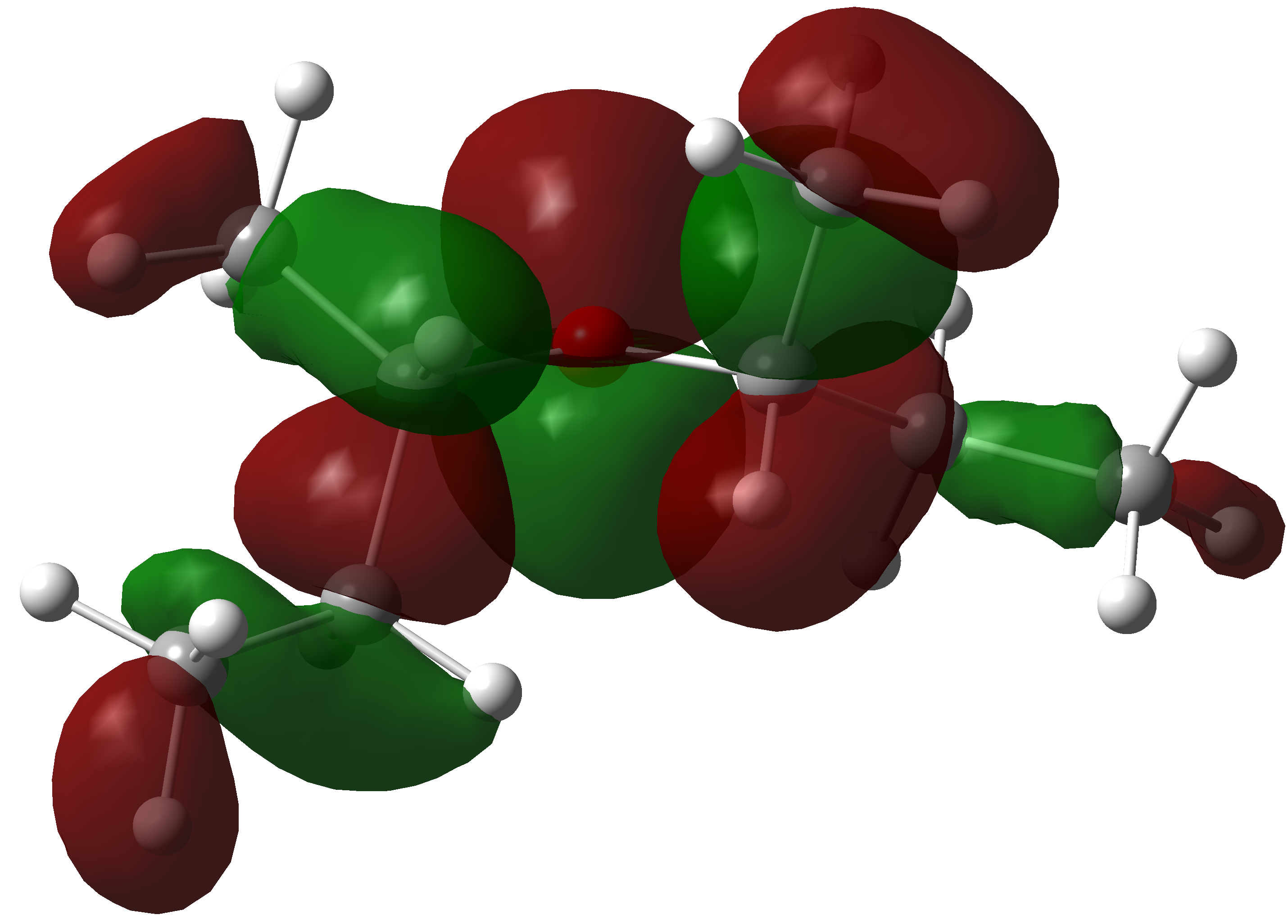 |
| LUMO | 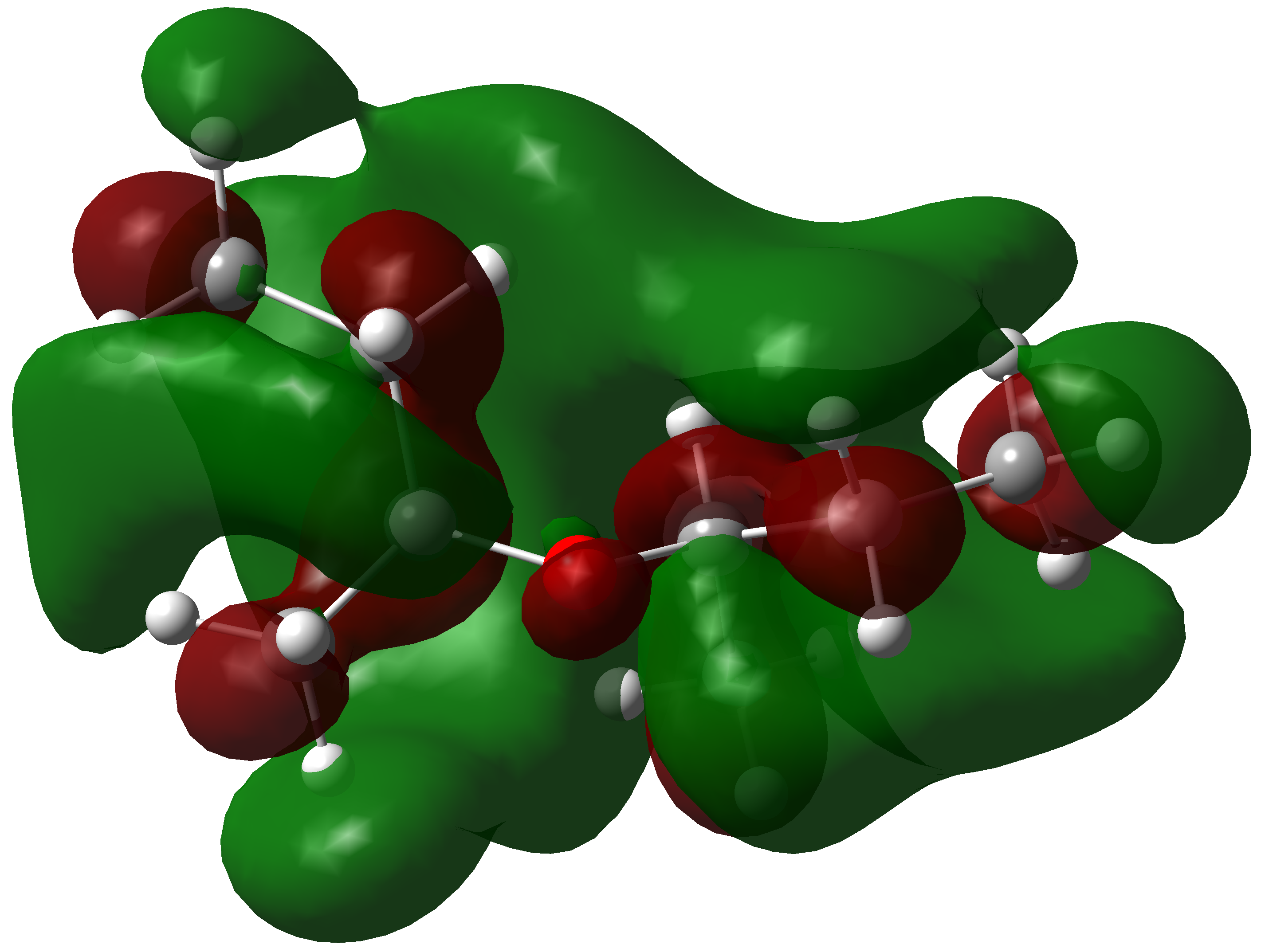 | 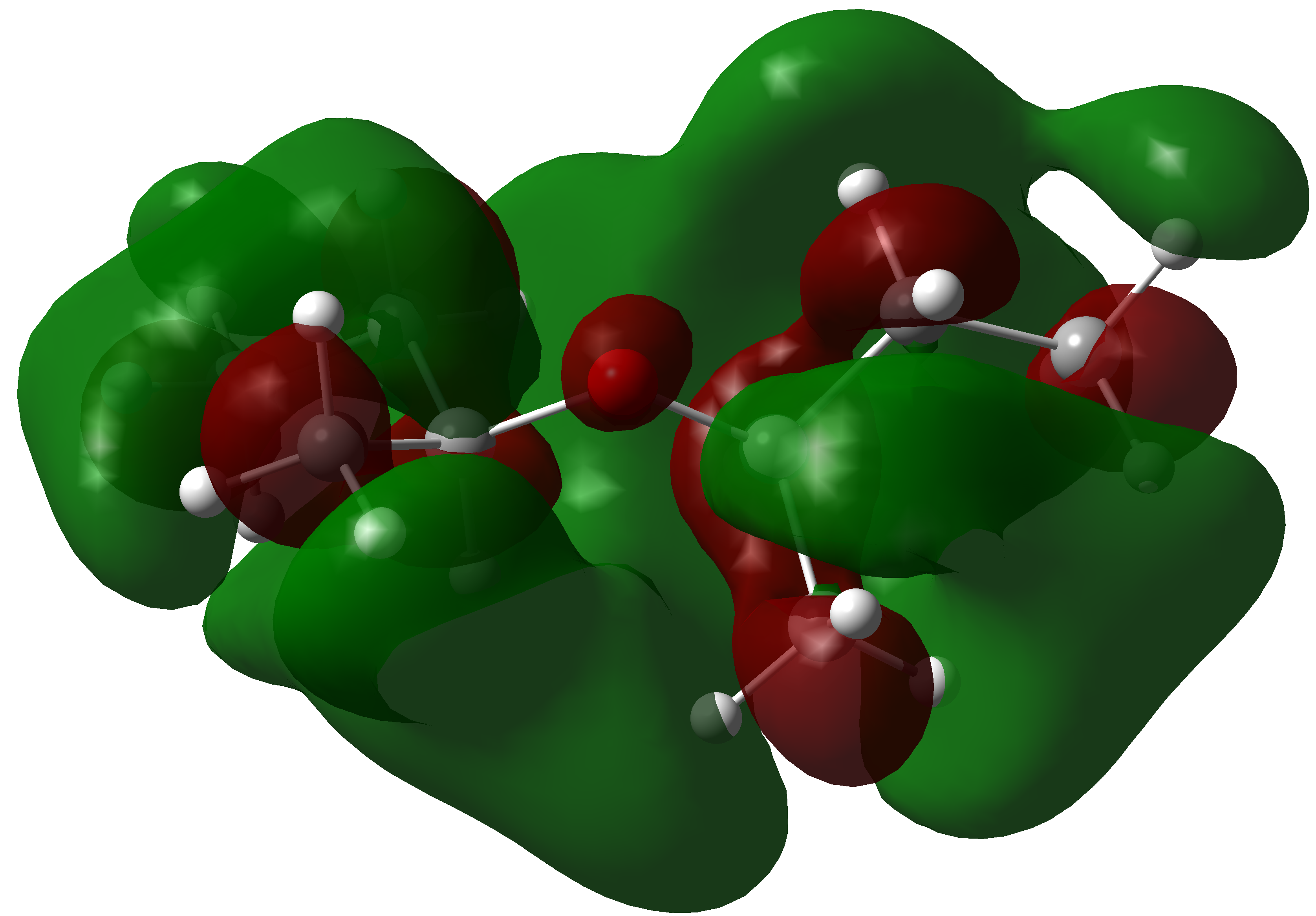 |

**FİGURE S1.** HOMO-LUMO Orbitals of di-sec-butyl ether in gas phase and methanol phase

|  | Gas | Methanol |
| --- | --- | --- |
| HOMO | 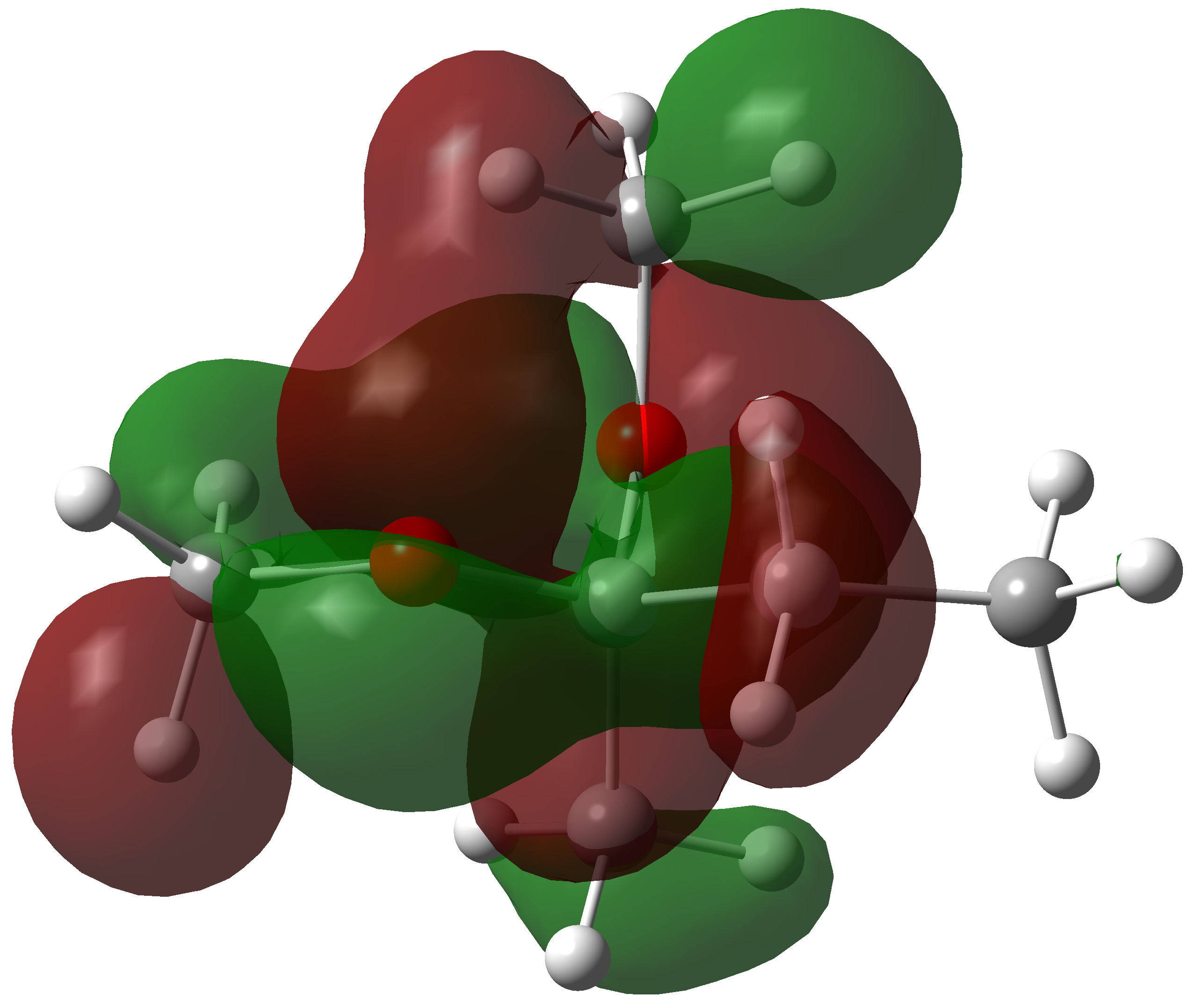 | 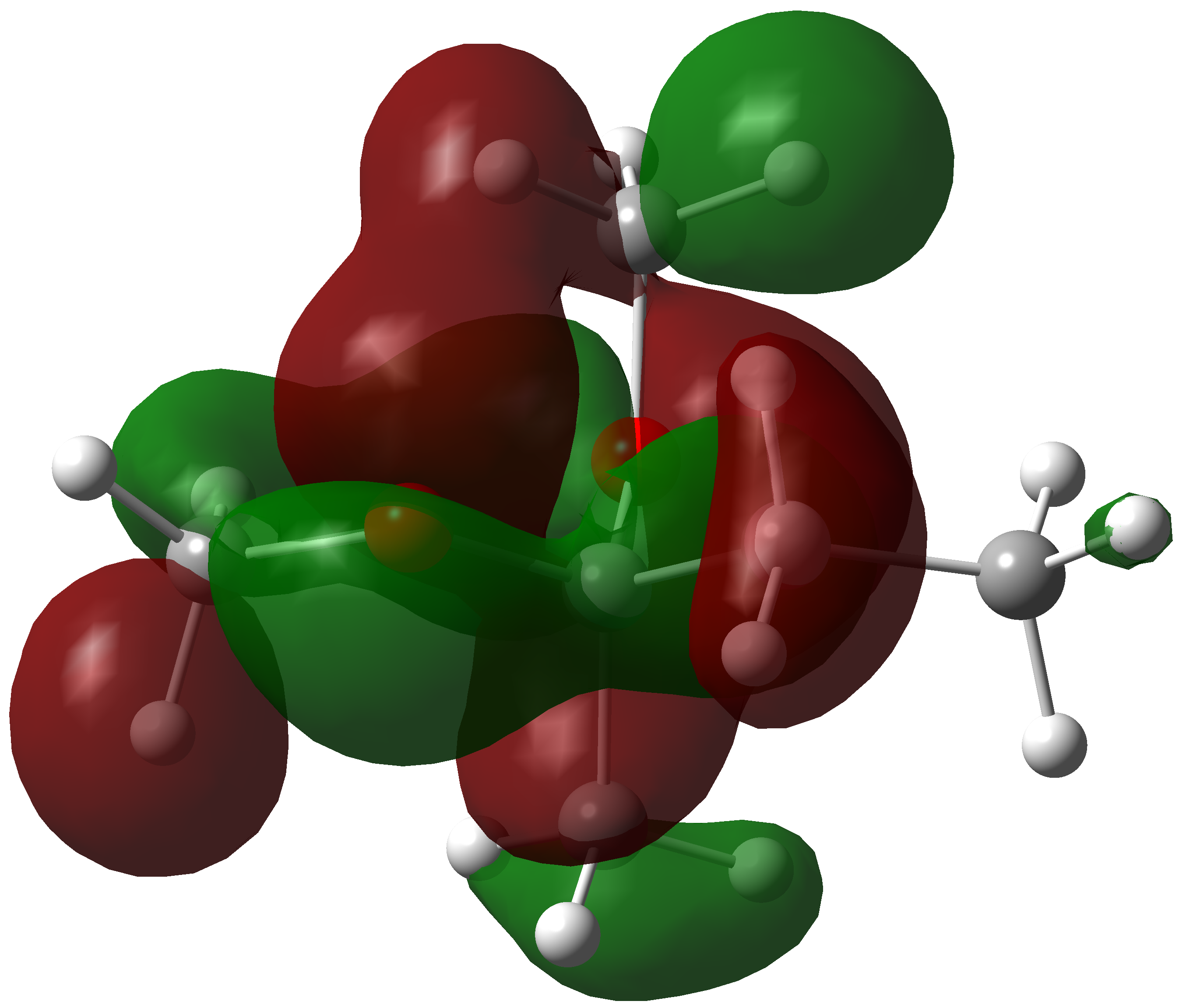 |
| LUMO | 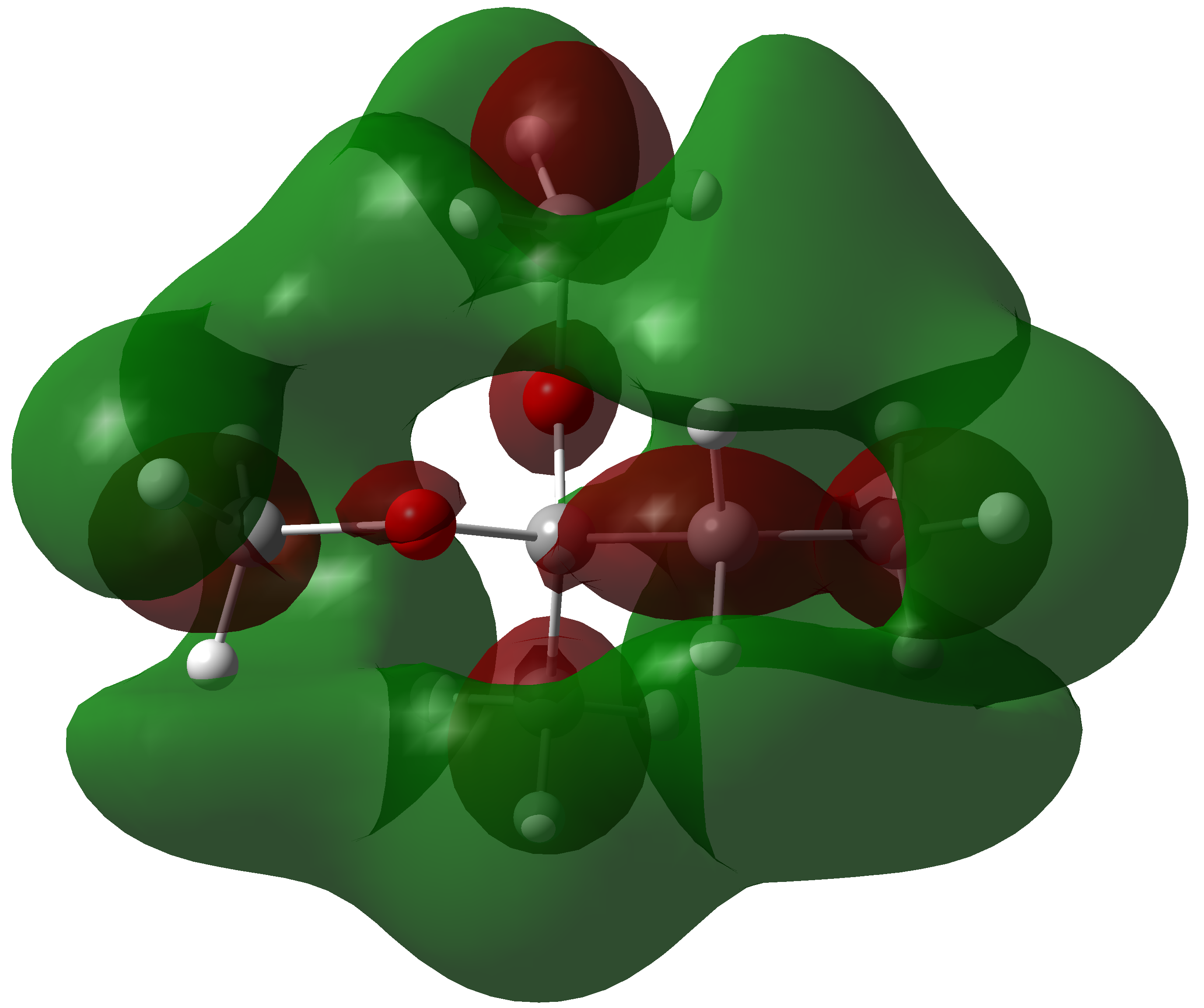 | 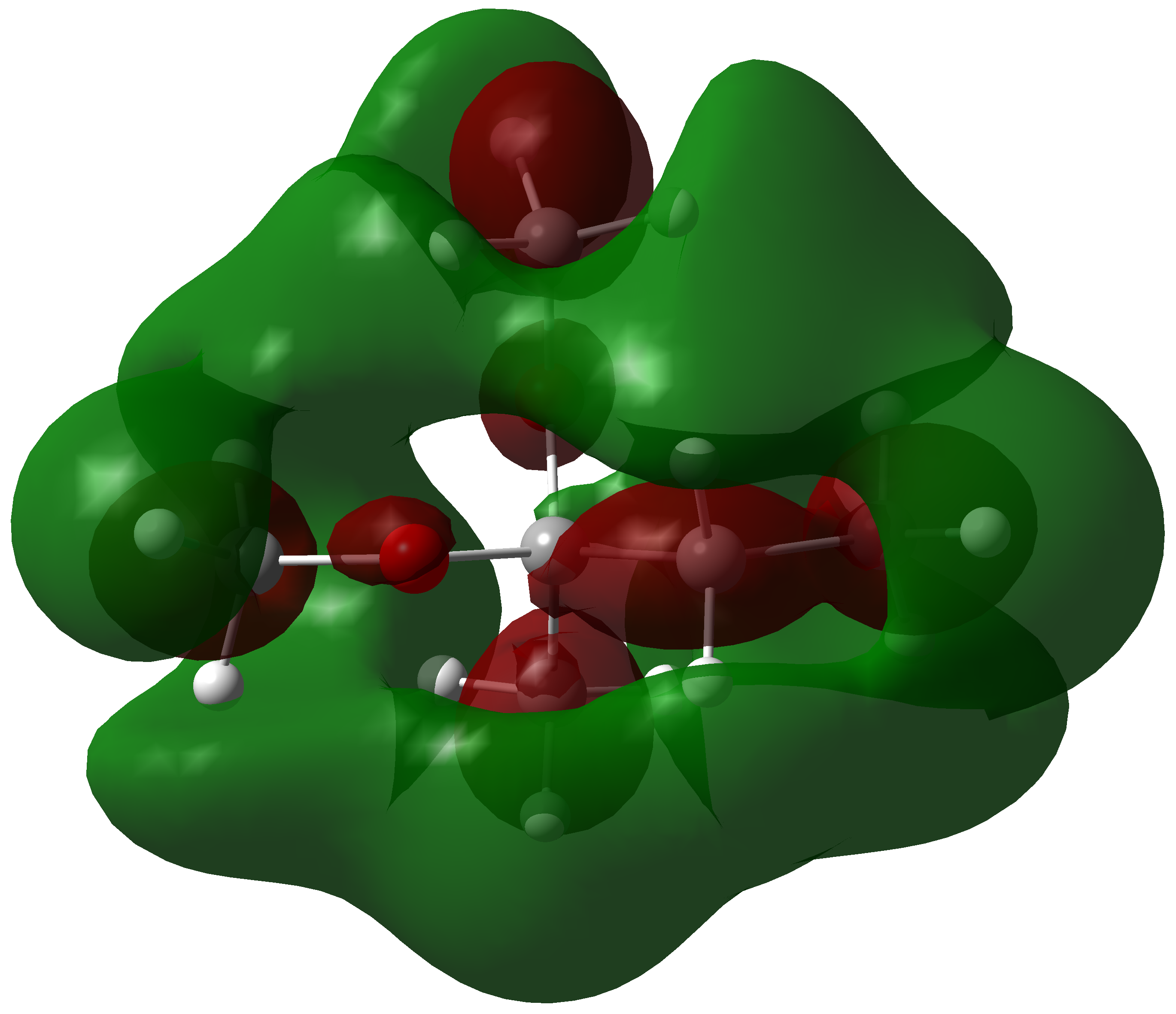 |

**FİGURE S2** HOMO-LUMO Orbitals of 2,2-dimethoxybutane in gas phase and methanol phase

|  | Gas | Methanol |
| --- | --- | --- |
| HOMO | 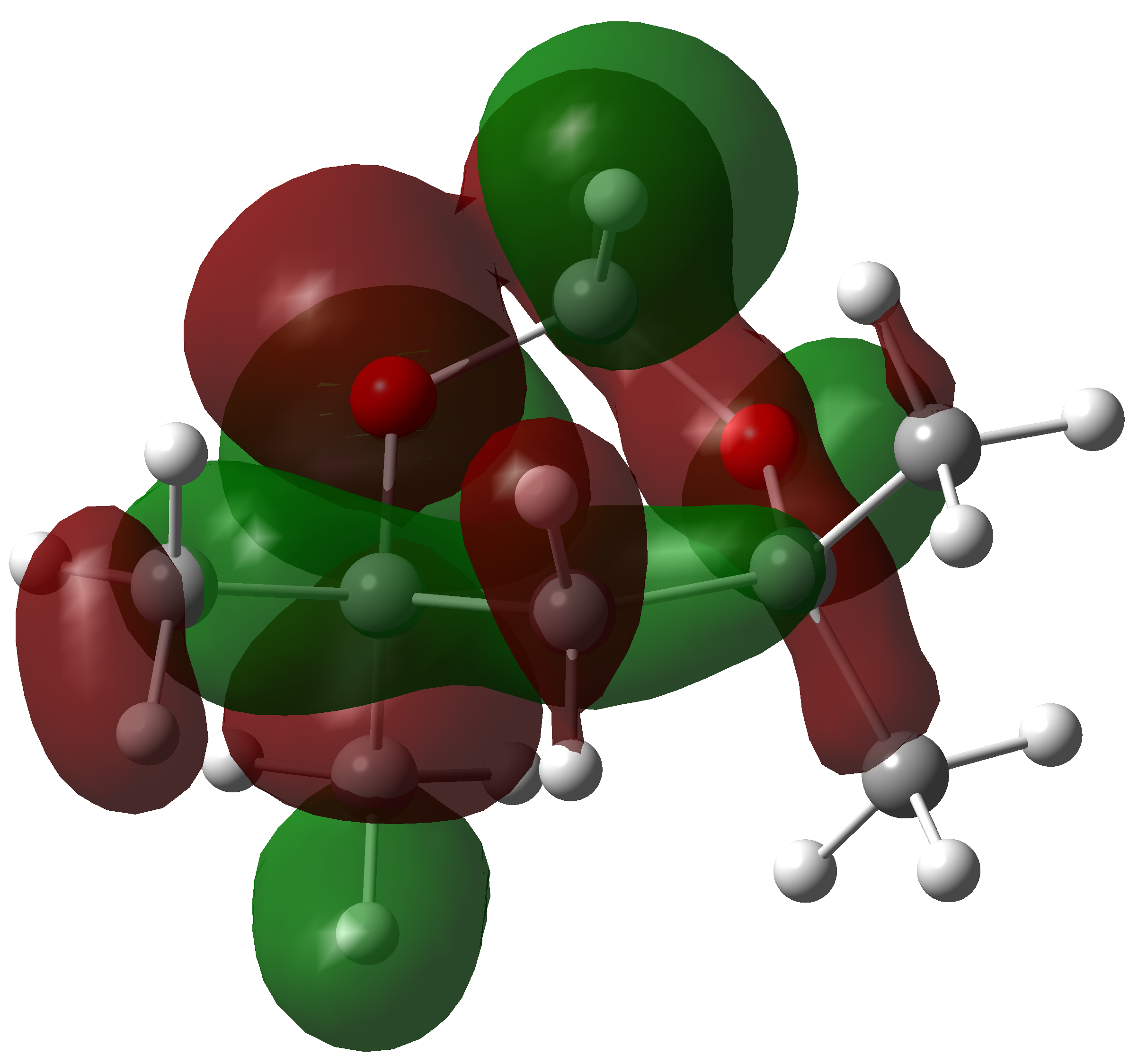 | 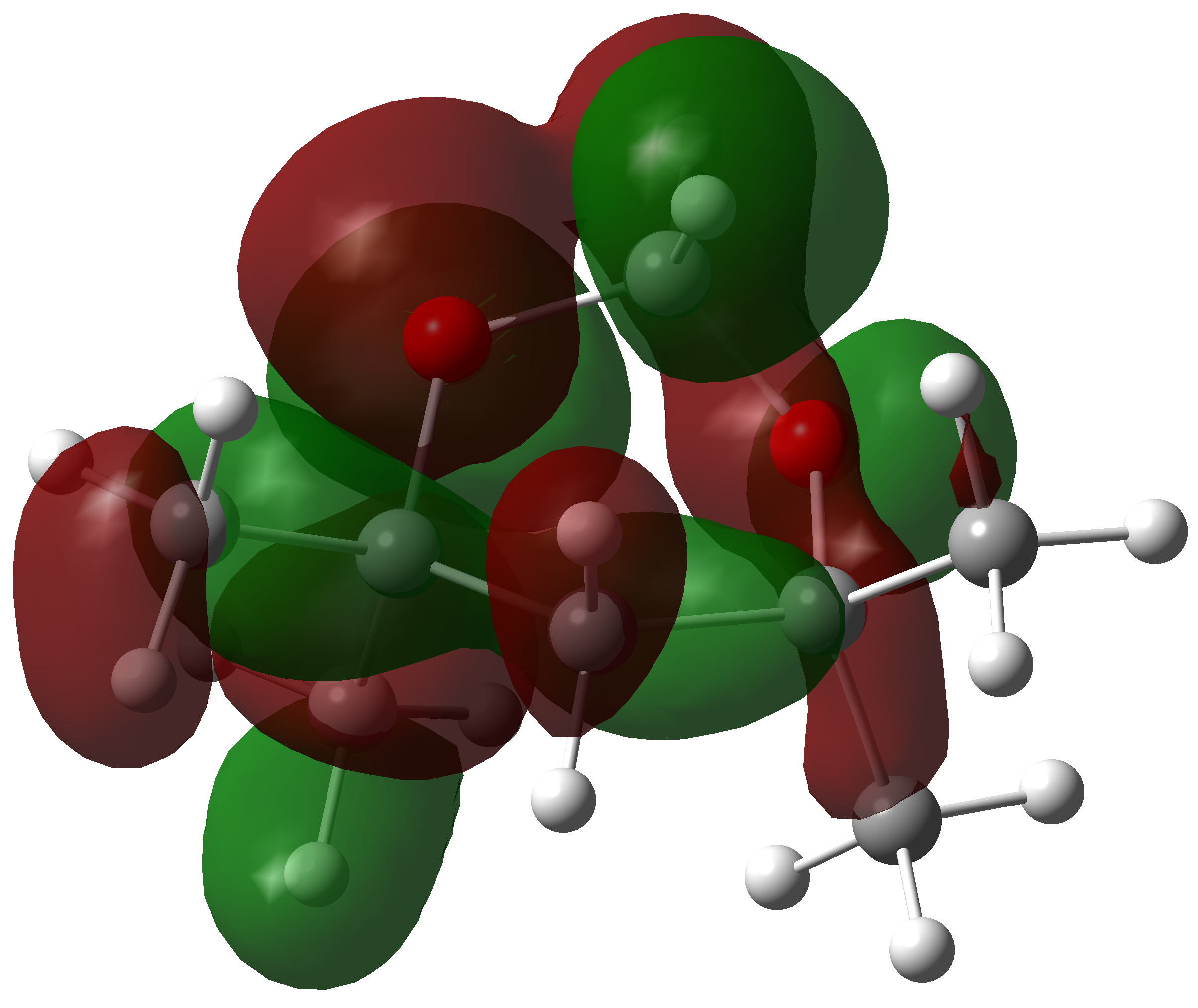 |
| LUMO | 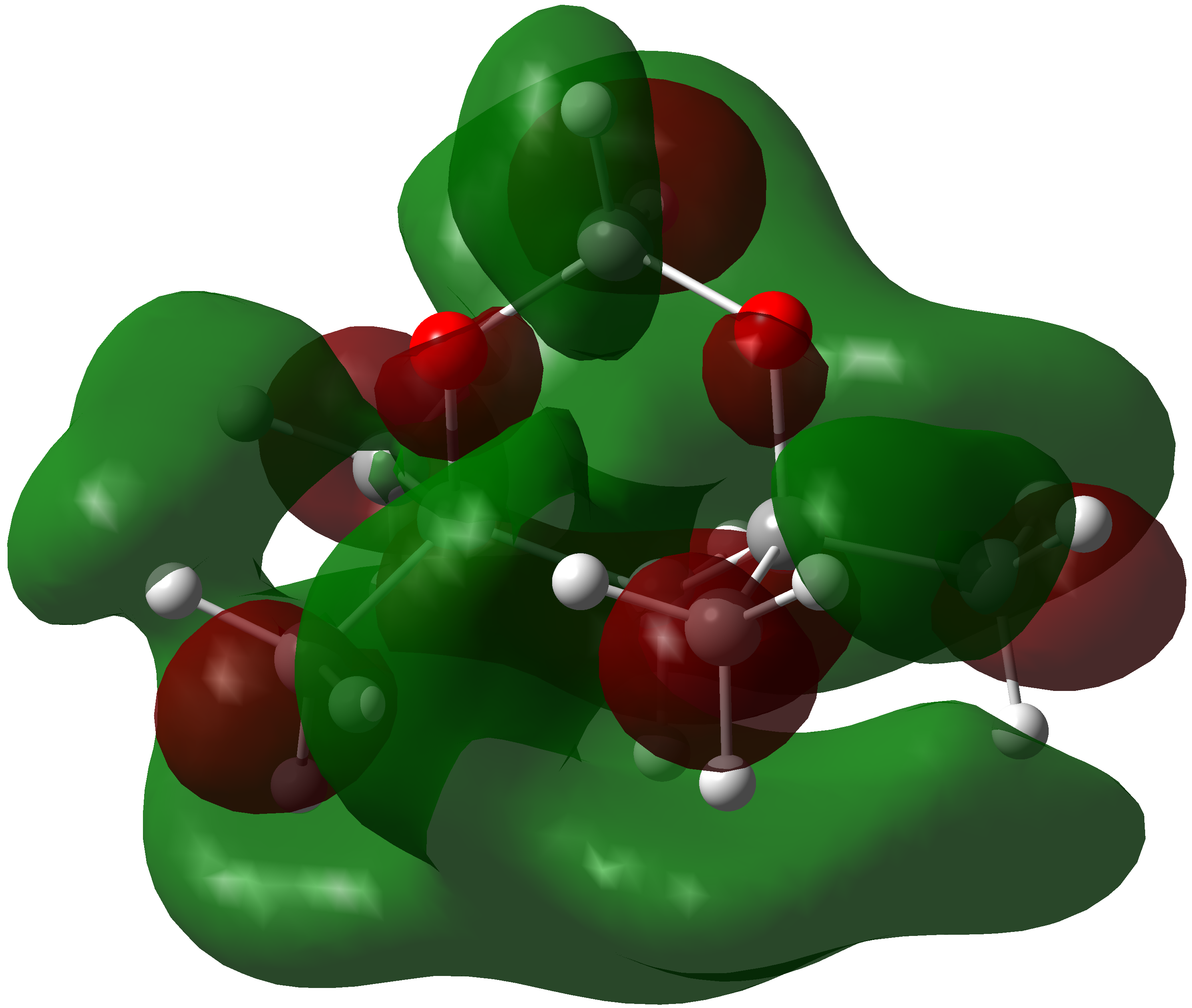 | 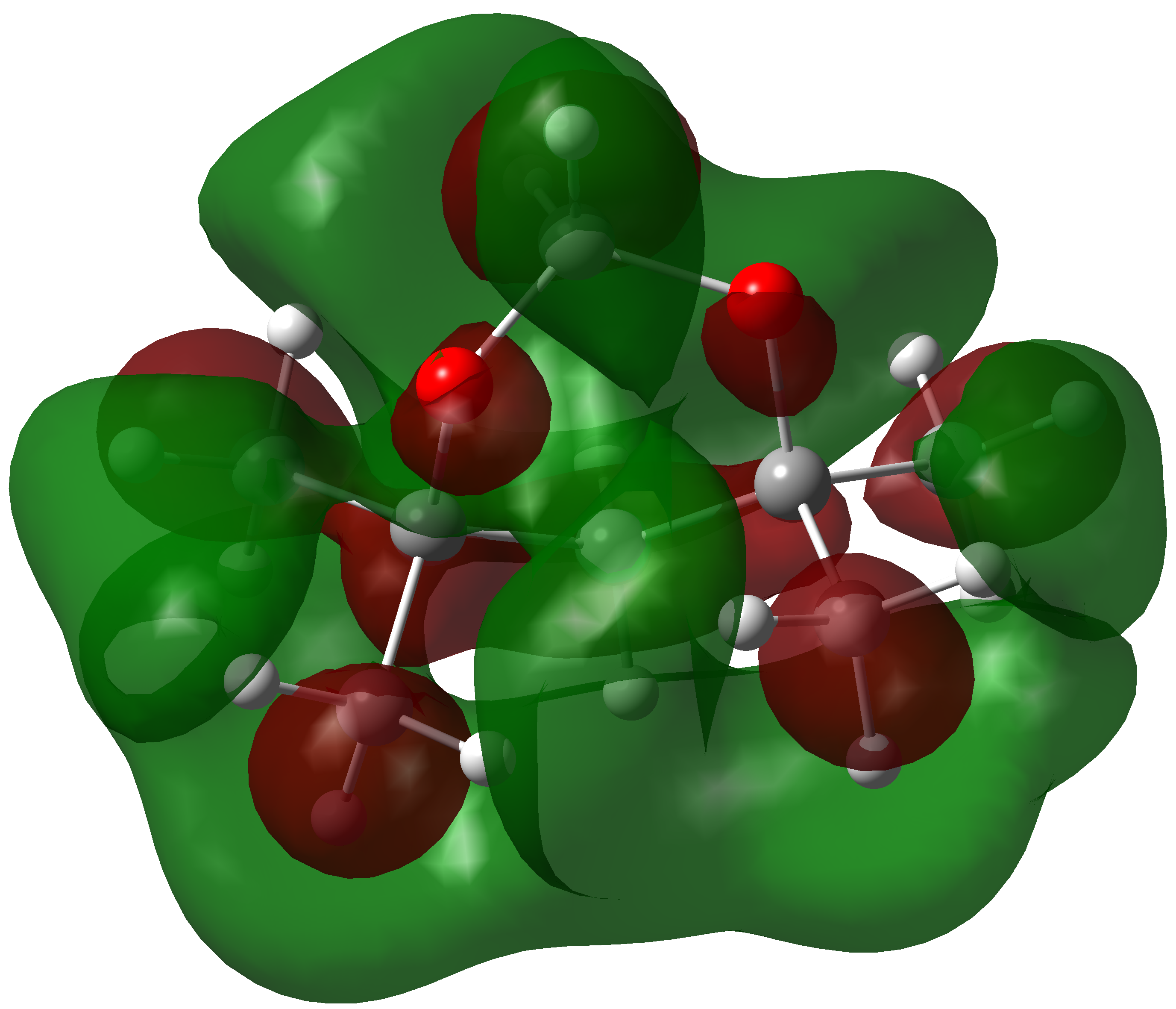 |

**FİGURE S3.** HOMO-LUMO Orbitals of 4,4,6,6-tetramethyl-1,3-dioxane in gas phase and methanol phase

|  | Gas | Methanol |
| --- | --- | --- |
| HOMO | 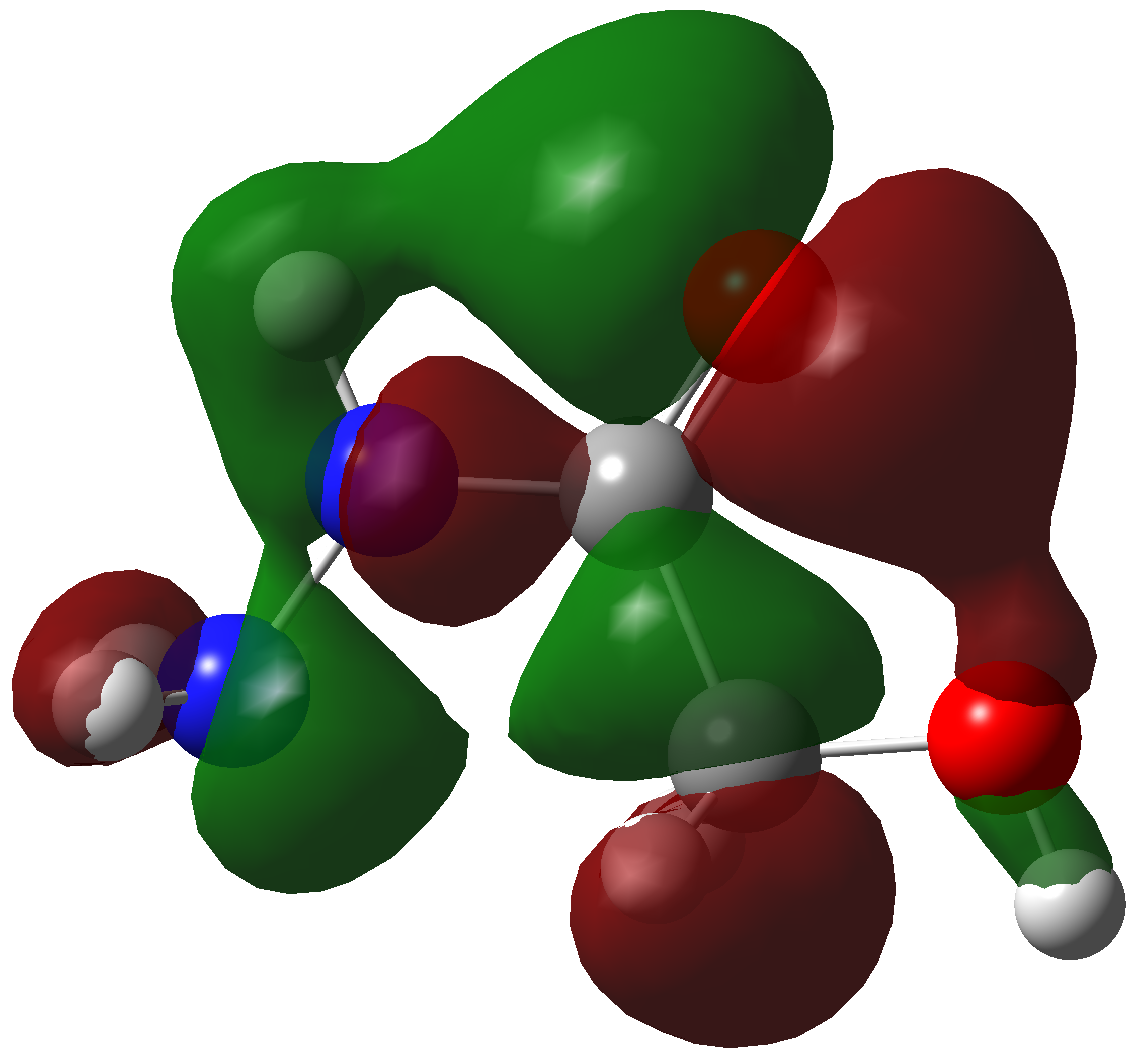 | 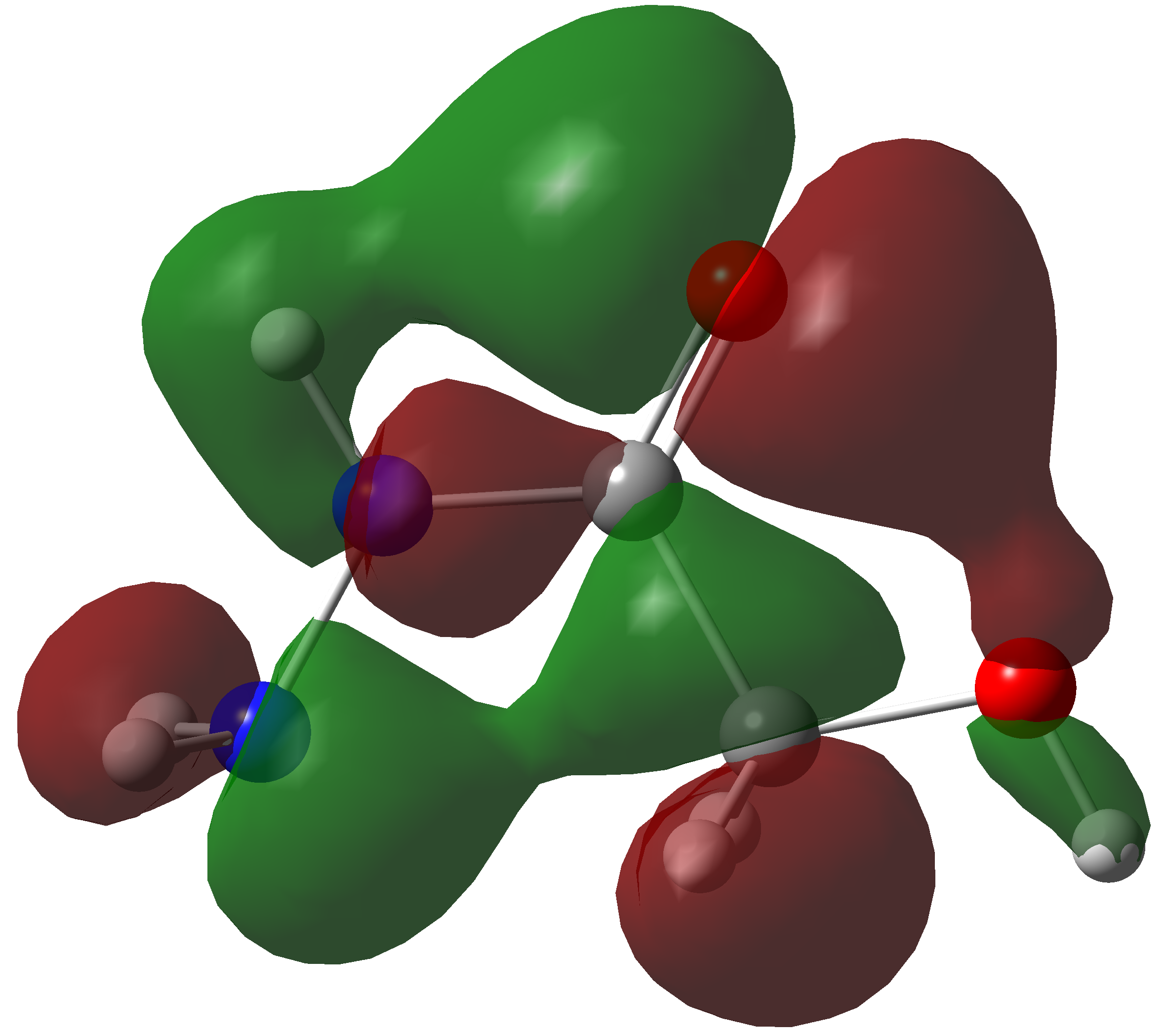 |
| LUMO | 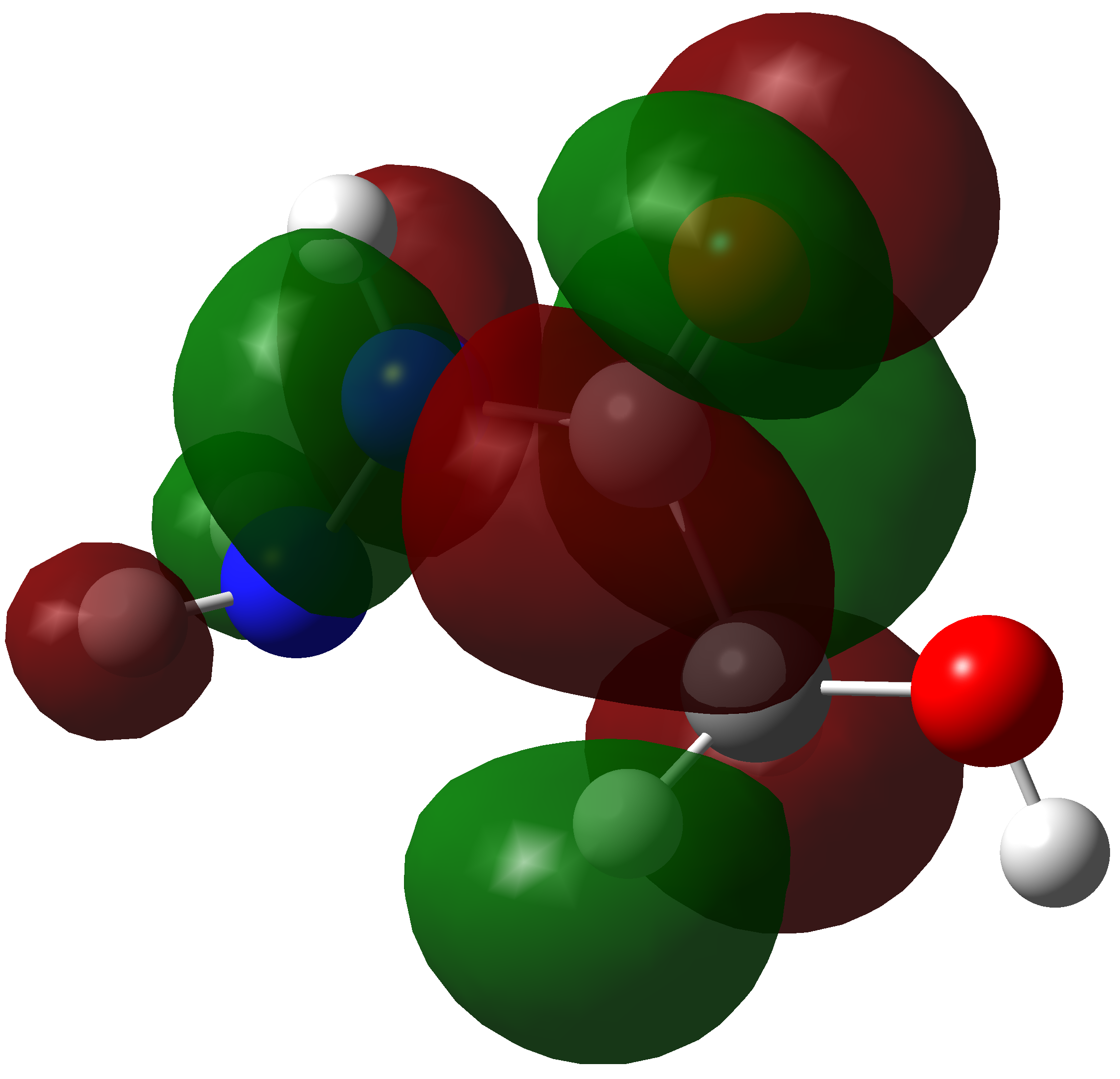 | 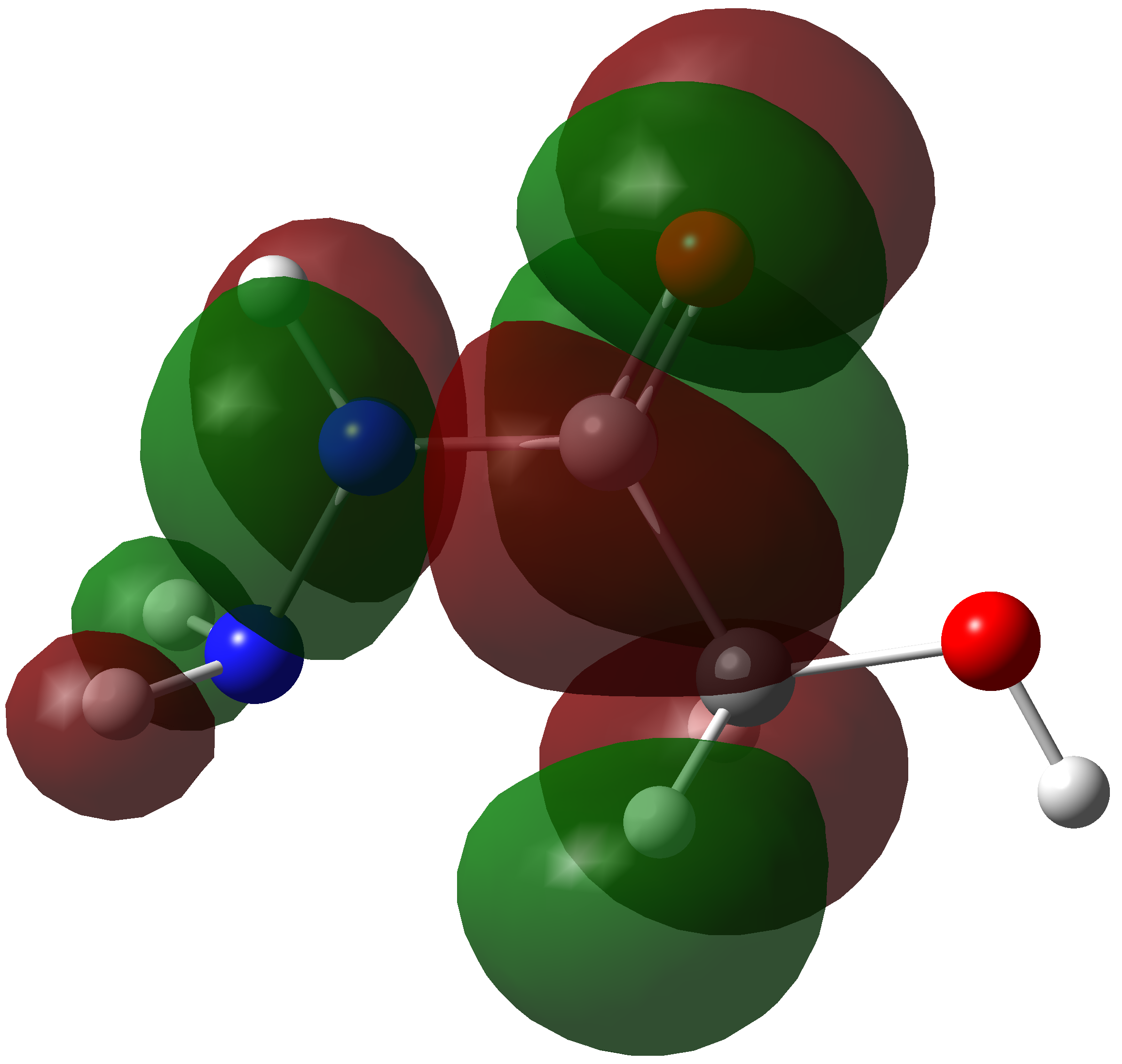 |

**FİGURE S4**. HOMO-LUMO orbitals of hydroxyacetic acid, hydrazide in gas phase and methanol phase

|  | Gas | Methanol |
| --- | --- | --- |
| HOMO | 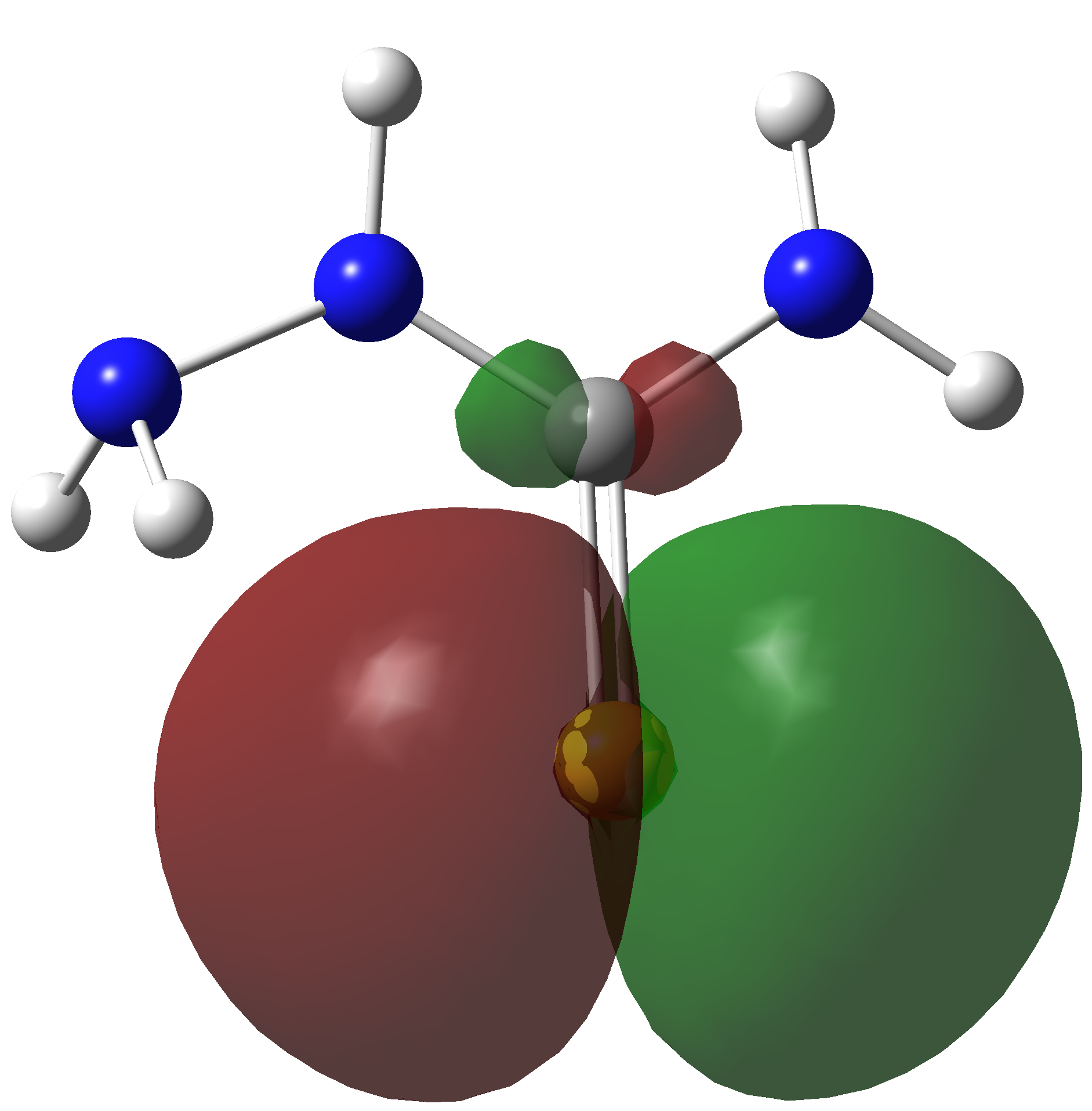 | 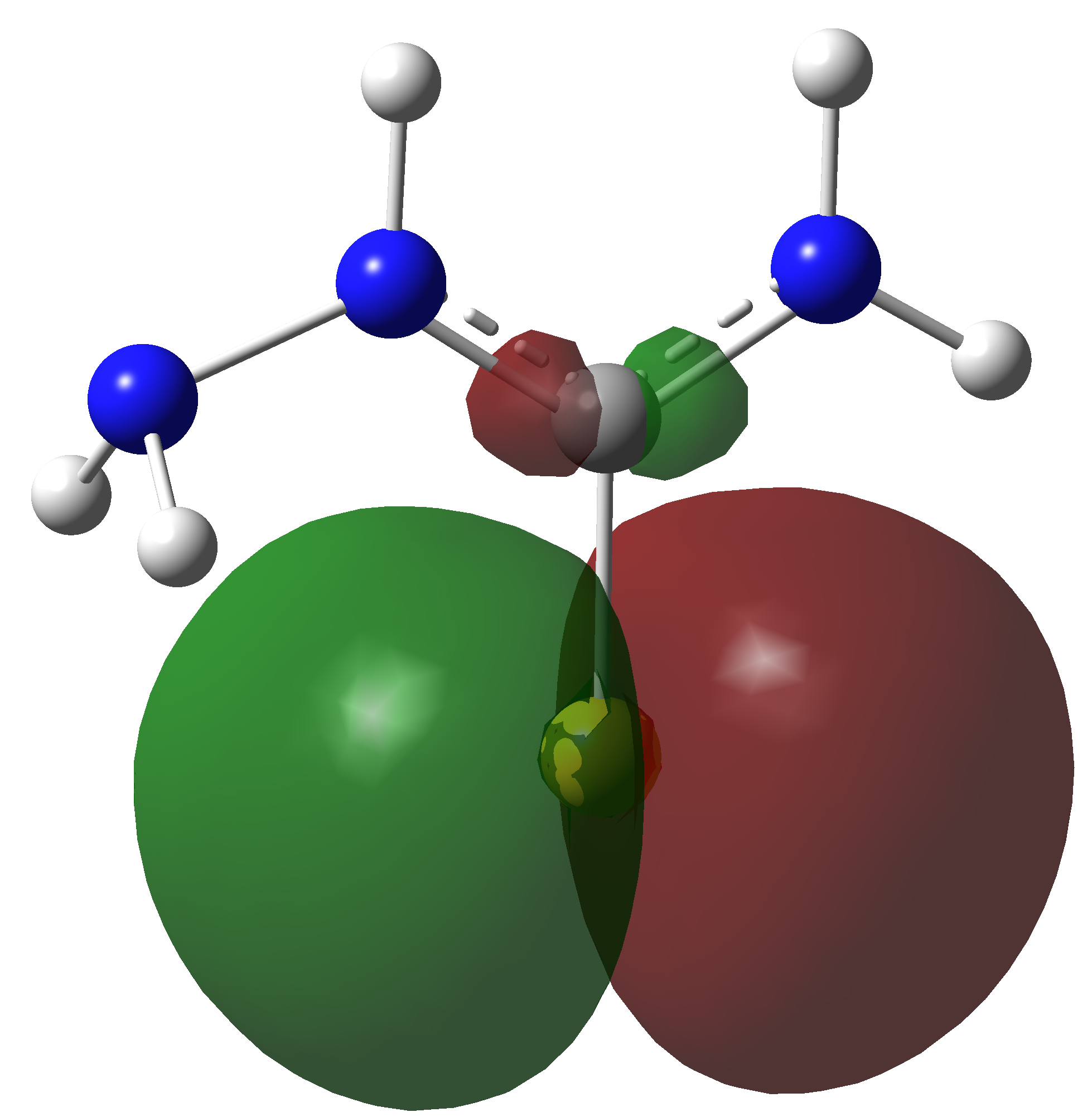 |
| LUMO | 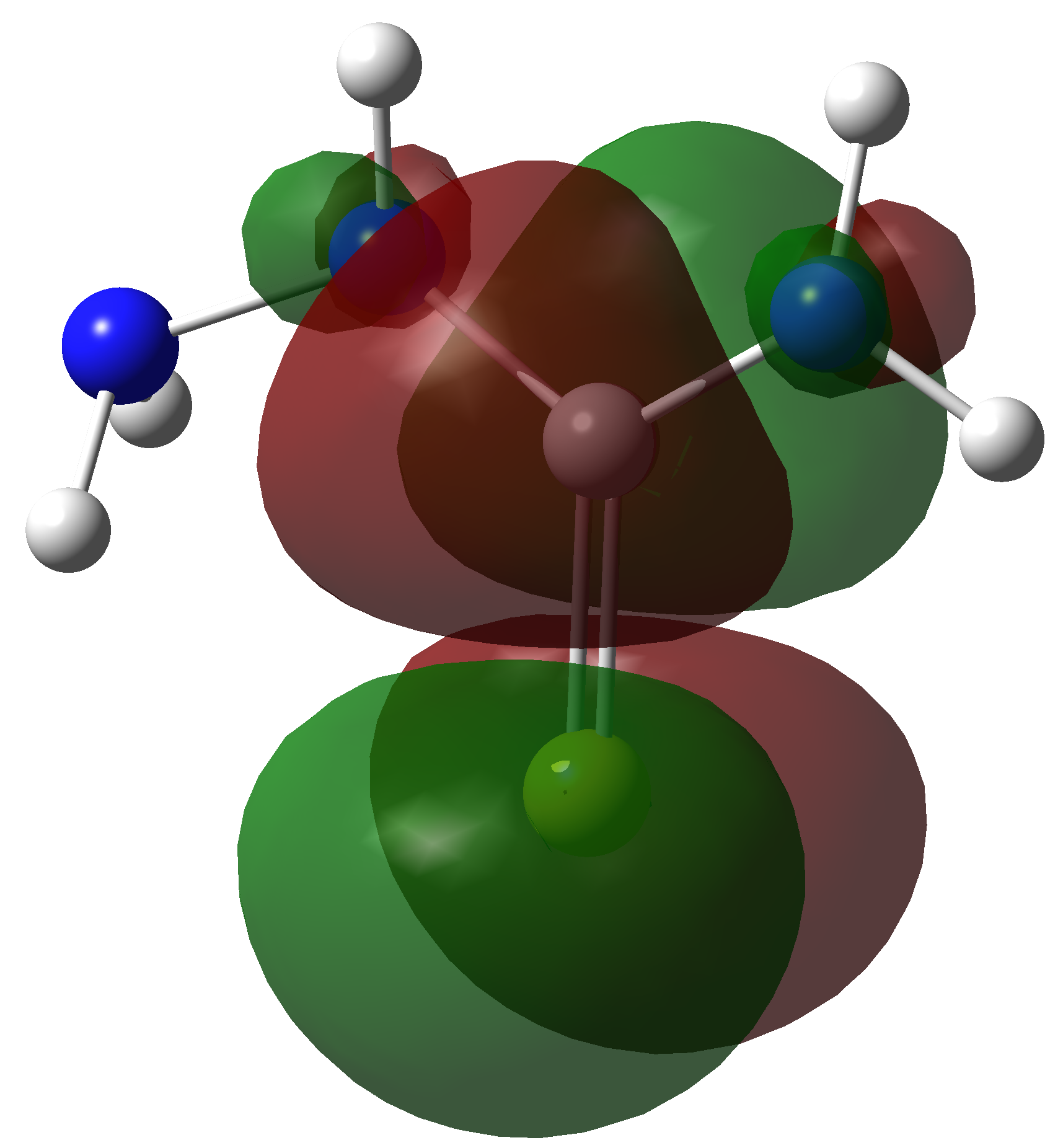 | 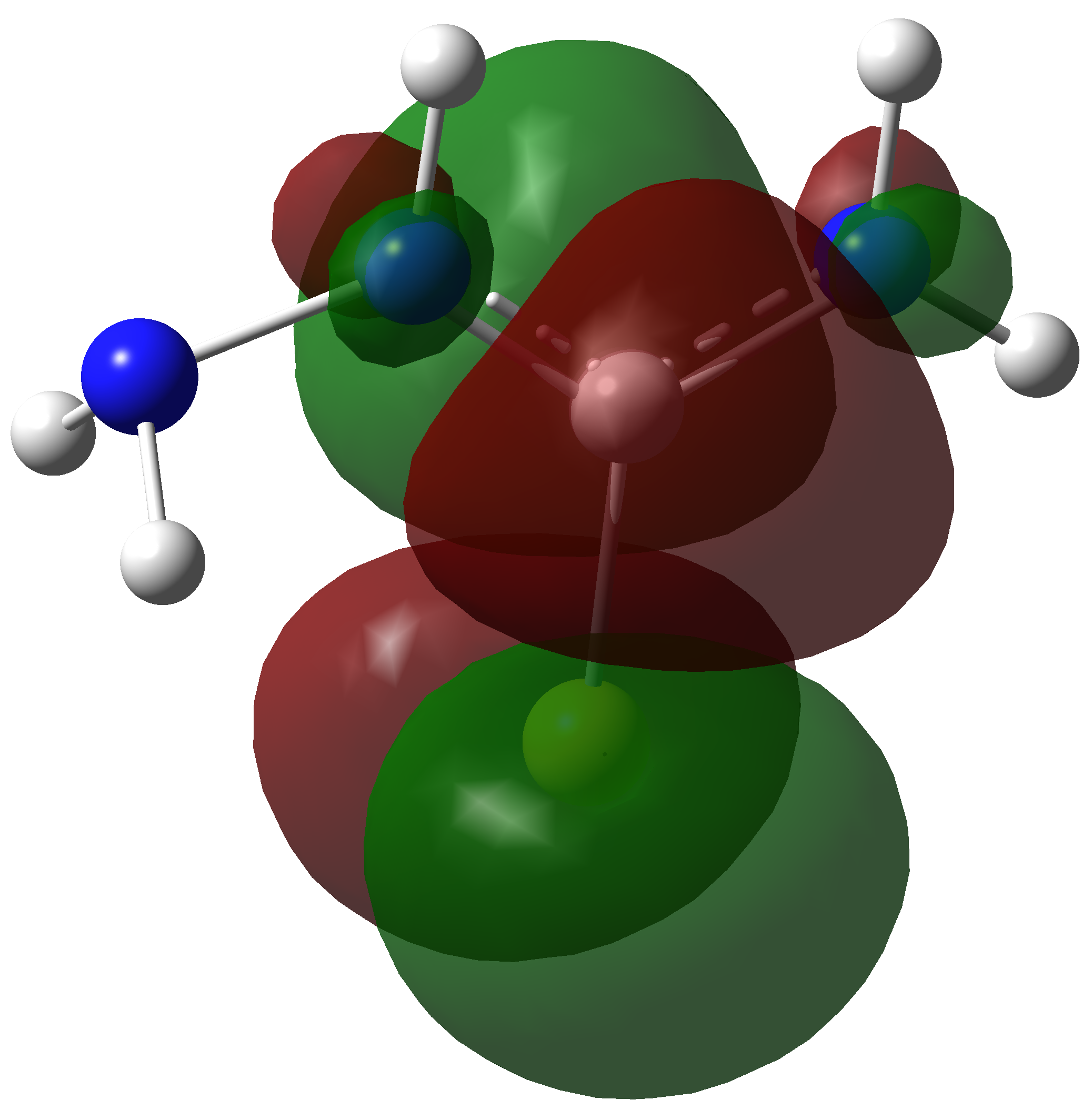 |

**FİGURE S5** HOMO-LUMO orbitals of hydrazinecarbothioamide in gas phase and methanol phase


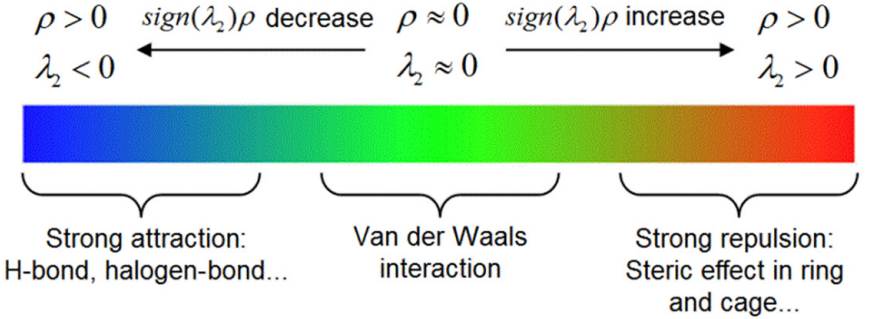


FİGURE S6. Classification of NCI using RDG isosurfaces with λ_2_​-ρ values and color representation

**TABLE S1.** Calculated HOMO-LUMO orbital energies, energy gaps (Egap), and global reactivity parameters of the compounds

| Compounds | Di-sec-butyl ether | | 2,2-Dimethoxybutane | | 4,4,6,6-Tetramethyl-1,3-dioxane | | Hydroxyacetic acid, hydrazide | | Hydrazinecarbothioamide | |
| --- | --- | --- | --- | --- | --- | --- | --- | --- | --- | --- |
|  | **gas** | **methanol** | **gas** | **methanol** | **gas** | **methanol** | **gas** | **methanol** | **gas** | **methanol** |
| E_HOMO_ | -6.513 | -6.623 | -6.984 | -7.104 | -6.599 | -6.760 | -6.578 | -6.905 | -5.234 | -5.616 |
| E_LUMO_ | 2.229 | 2.272 | 2.292 | 2.286 | 1.993 | 2.077 | 1.032 | 0.794 | -1.722 | -2.193 |
| Egap,ΔE | 8.742 | 8.895 | 9.276 | 9.390 | 8.592 | 8.837 | 7.610 | 7.699 | 3.512 | 3.423 |
| IP | 6.513 | 6.623 | 6.984 | 7.104 | 6.599 | 6.760 | 6.578 | 6.905 | 5.234 | 5.616 |
| EA | -2.229 | -2.272 | -2.292 | -2.286 | -1.993 | -2.077 | -1.032 | -0.794 | 1.722 | 2.193 |
| χ | 2.142 | 2.175 | 2.346 | 2.409 | 2.303 | 2.341 | 2.773 | 3.055 | 3.478 | 3.904 |
| μ | -2.142 | -2.175 | -2.346 | -2.409 | -2.303 | -2.341 | -2.773 | -3.055 | -3.478 | -3.904 |
| η | 4.371 | 4.447 | 4.638 | 4.695 | 4.296 | 4.418 | 3.805 | 3.849 | 1.756 | 1.711 |
| ζ | 0.114 | 0.112 | 0.108 | 0.106 | 0.116 | 0.113 | 0.131 | 0.130 | 0.284 | 0.292 |
| ω | 0.525 | 0.532 | 0.593 | 0.618 | 0.617 | 0.620 | 1.010 | 1.212 | 3.444 | 4.453 |
| N | 1.904 | 1.880 | 1.685 | 1.618 | 1.619 | 1.611 | 0.989 | 0.824 | 0.290 | 0.224 |
